# Regioselective Halogenation of 1,4-Benzodiazepinones via CH Activation

**DOI:** 10.1038/srep12131

**Published:** 2015-07-16

**Authors:** Hajer Abdelkafi, Jean-Christophe Cintrat

**Affiliations:** 1 CEA, iBiTecS, Service de Chimie Bioorganique et de Marquage, LabEx LERMIT. 91191 Gif-sur-Yvette (France)

## Abstract

This article reports an efficient CH activation process for regioselective halogenation of 1,4-benzodiazepinones. Direct halogenation with NXS (X = Br, I) affords halogenated benzodiazepinones on the central aromatic ring whereas catalyst (Pd(OAc)_2_) controlled CH activation furnishes regioselectively ortho halogenated benzodiazepinones on the phenyl side chain.

Over the past decade, Pd(II)-catalyzed halogenation of CH bond guided by an intramolecular directing group has emerged as an efficient method in organic synthesis. Different halogenating reagents and directing groups have been employed for the direct transformation of C-H bond to C-X (X = Cl, Br, I) bond^1^,^2^,^3^,^4^,^5^,^6^,^7^,^8^,^9^. During our studies on 1,4-benzodiazepinones as bioactive molecules on different targets^10^,^11^,^12^ we were interested in developing direct halogenation taking advantage of the internal ketimine to direct the CH activation. It has been proven that the presence of halogen atoms at the 2′ and/or 7 position, for 1,4-benzodiazepinones acting as GABA receptor allosteric modulators, result in increased receptor affinity^13^. Hence few marketed psychotropes are halogenated 1,4-benzodiazepinones (Fig. 1)^14^.

To the best of our knowledge, no direct introduction of halogen atoms at the 2′ position of the 1,4-benzodiazepinone skeleton has been described. Designing such a route from the already synthesized benzodiazepinones would allow a quick entry to halogenated analogues or functionalized derivatives via metal catalyzed cross-coupling reactions.

A direct CH activation of this ortho position could be a straightforward route to halogenated benzodiazepinones. We were encouraged in this approach based on recent reports that described the synthesis of 1,4-benzodiazepinones derived metallacycles with either zinc^15^, palladium^16^,^17^,^18^ or ruthenium^19^. Here we report a direct halogenation of 1,4-benzodiazepinones via a catalytic CH activation.

## Results and discussion

The palladium-catalyzed directed CH activation/halogenation reactions on model compounds usually proceed in good yield and with complete control of regioselectivity^9^. We therefore started with unsubstituted benzodiazepinone **1** to find the best experimental conditions to get halogenation of the C phenyl ring with minimum amount of side reaction on the A phenyl ring. Microwave heating was preferred to conventional heating to lower the reaction time and the amount of degradation compounds.

Few palladium catalysts were initially tested with NIS as halogenating agent but only palladium acetate afforded the halogenated compound albeit with low yield. Reaction performed in acetonitrile with 1.5 equivalents of NIS at 80 °C for 15 minutes afforded solely the monohalogenated compound **1-I** (Table 1, entry 1). Raising the reaction temperature to 100 °C fully converted the starting material but 15% of the 2′,6′-dihalogenated compound **1-II** was also observed (Table 1, entry 2). Upon warming to 120 °C dimerization of the starting material was observed along with degradation (Table 1, entry 3). Finally reaction at 100 °C without palladium catalyst never yielded iodination even with two equivalents of NIS and prolonged reaction time (Table 1, entries 4 and 5).

**Figure 2:**
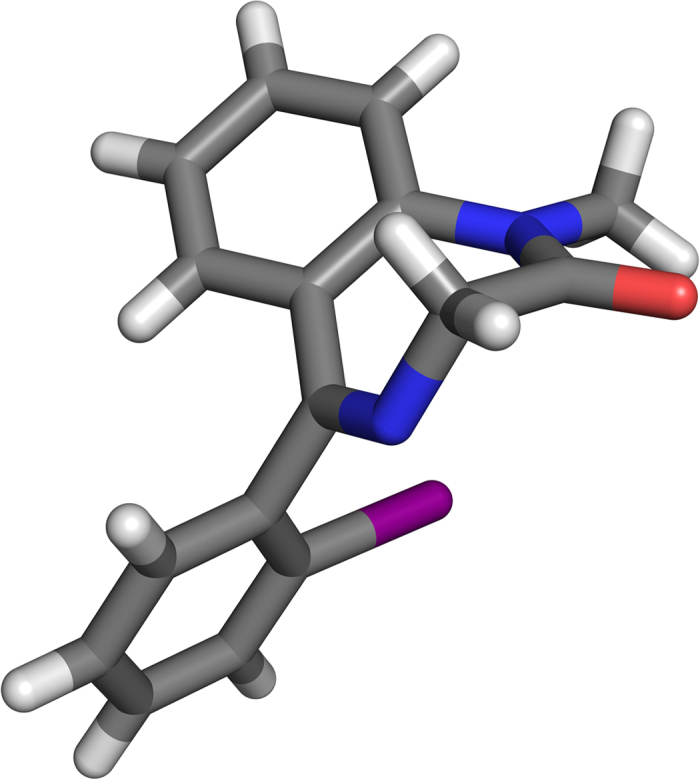
Crystal structure of compound 1-I.

The ortho position of the iodine was assigned by NMR analysis and unambiguously confirmed from X-Ray crystallography (see Fig. 2).

Surprisingly, a drop of reactivity was observed when NBS and NCS were used as halogenating agents. Using the previously optimized conditions in the presence of NBS gave less conversion to **1-Br** (50% of starting material left) along with some brominated compound on the central phenyl ring **2** (Table 1 entry 6). Increasing the amount of NBS to 5 equivalents, prolonged heating (1 hour) and replacing CH_3_CN by DMF resulted in more compound **2** (Table 1, entry 7). Interestingly, reaction performed without palladium acetate only resulted in compound **2** (Table 1, entry 8). To the best of our knowledge, no direct halogenation of 1,4-benzodiazepinone on the 7-position was reported (compound **2**). The 7-bromo position was unequivocally identified by comparison with NMR spectra of 7-bromo-1,3-dihydro-5-phenyl-2H-1,4-benzodiazepin-2-one obtained from 2-amino-4-bromobenzophenone. This regioselective control affords a straightforward access to 7-brominated benzodiazepinones. Replacing NBS by another brominating agent, CuBr_2_, gave the starting material unchanged (Table 1, entry 9). Reaction with NCS at 100 °C in CH_3_CN only afforded the mono-chlorinated compound **1-Cl** as traces whereas the same reaction without palladium acetate in DMF only yielded untractable mixture of monochlorinated compounds.

Having the best experimental conditions (NIS, palladium acetate, MW heating at 100 °C for 15 minutes) in hand, we decided to check the scope and limitation of this iodination process on differently substituted benzodiazepinones (Table 2). Each starting material was synthetized from the corresponding aminobenzophenones^20^,^21^.

It is worth noting that radioiodinated (^123^I, ^125^I or ^131^I) benzodiazepinones have been widely used in binding assays^13^. The strategies used in the synthesis of these radioiodinated benzodiazepinones always involve an isotopic exchange with Na^125^I, the parent iodinated compound being synthesized from simple iodinated precursors such as iodobenzoic acid^22^,^23^,^24^.

Our previously optimized iodinating conditions (Table 2, entry 1) allowed a clean conversion of the 7-bromo compound **2** into the monoiodinated compound **2-I** with 61% yield along with 3% of the diiodinated compound **2-II** (Table 2, entry 2). Notably, when one ortho position of the ring was already substituted slightly lower yield was experienced (Table 2, entry 3). Introduction of a methyl group on the 4′ position does not alter the course of the reaction giving also fair yield and had no impact of the ratio of mono and diiodinated compounds **4-I** and **4-II** (Table 2, entry 4). Interestingly, the iodination also proceeds satisfactorily in the presence of a secondary amide (no methyl group on nitrogen 1) (Table 2, entry 5) even if the use of a benzyl protecting group allows the formation of the desired iodinated compound **7-I** with a better yield (Table 2, entry 7). Application of this protocol to a drug was also envisioned. Therefore diazepam was submitted to NIS/Pd(OAc)_2_ and clean conversion to the mono-iodinated analog **6-I** (Table 2, entry 6) was also successfully observed. It is worth precising here that this iodinated derivative showed higher receptor affinity than diazepam (IC_50_ 0.74 nM versus 6.70 nM)^22^. Finally, the 4′-methylated analog afforded comparable yield and ratio of **8-I** and **8-II** compounds compared to unsubstituted benzodiazepinones (Table 2, entry 8). A copy of ^1^H and ^13^C NMR spectra of all new compounds are available in the supplementary material from page S2 to S17.

**Figure 3:**
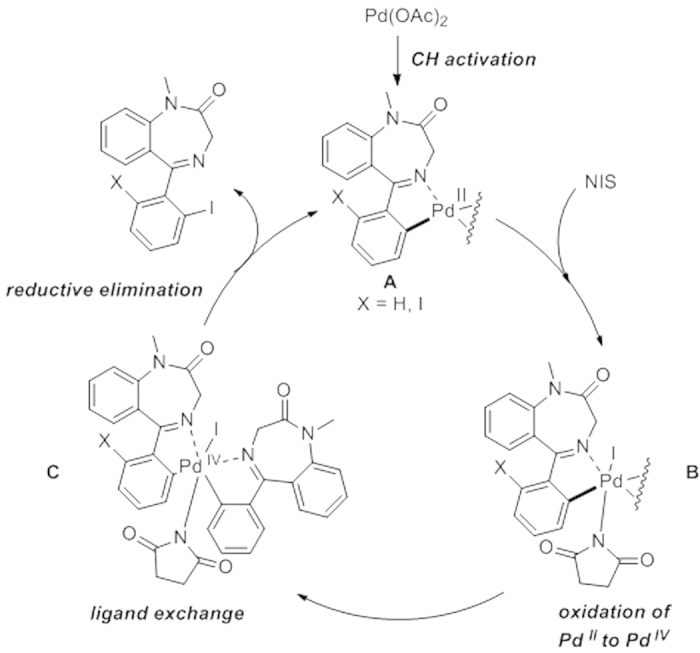
Proposed CH activation based mechanism of benzodiazepinones iodination with Pd(OAc)_2_.

The putative mechanism involves a CH activation assisted by the chelation of the nitrogen atom of the imino moiety of the benzodiazepinone. The insertion of a molecule of N-iodosuccinimide^25^ allows the formation of complex **B** on which the Pd(II) was oxidized to Pd(IV). Then, ligand exchange provides dimeric intermediate **C** whose formation was confirmed by LC-MS. Finally, a reductive elimination occurs to afford mono-iodinated compound that can enter a new catalytic cycle to afford the diiodinated product (Fig. 3).

In this mechanism, we suggest that the oxidation of Pd(II) to Pd(IV) is prior to the ligand exchange step because the dimer formation, via a homoleptic complex, was not detected when benzodiazepine **1** was treated with Pd(OAc)_2_ in the absence of NIS. Once we suggested the formation of complex **C** we investigated whether the dimer would be formed from a reductive elimination or a simple homocoupling from the iodinated compound. For this purpose the same reaction was performed starting from compound **1-I** and only non-iodinated dimer was formed strongly supporting the homocoupling process^26^,^27^,^28^.

## Conclusions

In conclusion, we have described herein an efficient protocol for regioselective halogenation of benzodiazepinones. This protocol takes advantage of CH activation with palladium acetate. Since radio-iodinated NIS is easily prepared, the CH activation described here offers a straightforward route to radioiodinated benzodiazepinones. Halogenated benzodiazepinones also represent ideal starting materials for more functionalized analogs via metal catalyzed cross-coupling reactions and this CH activation based approach opens new possibilities in this area. In this case, sequential halogenation/coupling procedures at the 7 then at the 2′ position or vice versa could gain libraries of benzodiazepinones.

## Methods

### General remarks

The products were isolated by flash silica gel column chromatography (0.040–0.063 mm). Reactions were run without exclusion of air/moisture in a microwave tube. Reactions were monitored by NMR. ^1^H and ^13^C NMR spectra were recorded using a Bruker Avance 400 MHz Ultrashield spectrometer in CD_3_CN for all compounds except for compound **5-I** whose NMR analysis were conducted in (CD_3_)_2_SO for solubility reasons. The following abbreviations are used in reporting NMR data: s, singlet; d, doublet; t, triplet; q, quartet; dd, doublet of doublets; ddd, doublet of doublets of doublets; d appt, doublet of apparent triplet; m, multiplet. Infrared spectra were recorded on an FT-IR spectrophotometer. HPLC-MS analysis and purification were performed using a Waters system (2525 binary gradient module, in-line degasser, 2767 sample manager, 2996 Photodiode Array Detector) with a binary gradient solvent delivery system. This system was coupled with a Waters Micromass ZQ system with a ZQ2000 quadrupole analyzer. The ionization was performed by electrospray and the other parameters were as follows: source temperature 120 °C, cone voltage 20 V, and continuous sample injection at 0.3 mL∕ min flow rate. Mass spectra were recorded in both positive and negative ion mode in the m/z 100–2,000 range and treated with the Mass Lynx 4.1 software. High-resolution mass spectrometry (HRMS) was performed using the Imagif platform (CNRS, Gif-sur-Yvette, France), and recorded on ESI/TOF LCP premier XE mass spectrometer (Waters) using flow injection analysis mode.

### 2′-iodo-1,3-dihydro-1-methyl-5-phenyl-2H-1,4-benzodiazepin-2-one (1-I)

To a solution of 1,3-dihydro-1-methyl-5-phenyl-2H-1,4-benzodiazepin-2-one **1** (30 mg, 0.12 mmol) in CD_3_CN (1.2 mL), were added Pd(OAc)_2_ (2.7 mg, 0.012 mmol) and N-iodosuccinimide (54 mg, 0.24 mmol). The mixture was stirred at 100 °C under microwave irradiation for 15 minutes. The crude mixture was evaporated, diluted in ethyl acetate (10 mL), and washed with a 2 M aqueous solution of NaOH (5 mL). The residue was purified by flash chromatography (cyclohexane/ethylacetate 1:1) affording 30 mg (69%) of **1-I** and 2 mg (3%) of **1-II**. IR (NaCl, cm^−1^) 3057, 2988, 2850, 1676, 1611, 1573, 1489, 1449, 1361, 1324, 1280, 1201, 1167, 1128, 1076, 1046, 1014, 984, 939, 915; ^1^H NMR (400 MHz, CD_3_CN) δ 7.83 (d, *J* = 8.0 Hz, 1H, 3′-H), 7.56 (d appt, *J* = 1.5 Hz, *J* = 8.7 Hz, 1H, 8-H), 7.51-7.45 (m, 2H, 5′-H and 6′-H), 7.42 (d, *J* = 8.2 Hz, 1H, 9-H), 7.15 (ddd, *J* = 9.2 Hz, *J* = 6.4 Hz, *J* = 2.8 Hz, 1H, 4′-H), 7.09 (d appt, *J* = 7.9 Hz, *J* = 0.9 Hz, 1H, 7-H), 6.94 (dd, *J* = 7.8 Hz, *J* = 1.4 Hz, 1H, 6-H), 4.58 (d, *J* = 10.6 Hz, 1H, 3a-H), 3.76 (d, *J* = 10.6 Hz, 1H, 3b-H), 3.39 (s, 3H, 1-CH_3_); ^13^C NMR (100 MHz, CD_3_CN) δ 173.5 (C = N), 170.1 (C = O), 145.5 (C-1′), 140.3 (C-3′), 132.5 (C-8), 131.6 (C-4′ and C-5′ or C-6′), 130.1 (C-5a), 129.6 (C-6), 129.3 (C-6′ or C-5′), 124.9 (C-7), 122.6 (C-9), 118.3 (C-9a), 96.8 (C-2′), 57.7 (C-3), 35.1 (1-CH_3_); HR-MS (ESI+) m/z Calcd for C_16_H_14_IN_2_O [M+H^+^] 377.0151, Found 377.0145.

### 7-bromo-2′-iodo-1,3-dihydro-1-methyl-5-phenyl-2H-1,4-benzodiazepin-2-one (2-I)

To a solution of 7-bromo-1,3-dihydro-1-methyl-5-phenyl-2H-1,4-benzodiazepin-2-one **2** (16 mg, 0.048 mmol) in CD_3_CN (500 μL), were added Pd(OAc)_2_ (1,1 mg, 0.0048 mmol) and N-iodosuccinimide (22 mg, 0.096 mmol). The mixture was stirred at 100 °C under microwave irradiation for 15 minutes. The crude mixture was evaporated, diluted in ethyl acetate (10 mL), and washed with a 2 M aqueous solution of NaOH (5 mL). The residue was purified by flash chromatography (cyclohexane/ethylacetate 1:1) affording 12 mg (54%) of **2-I** and 2 mg (7%) of **2-II** IR (NaCl, cm^−1^) 3059, 2985, 2920, 2853, 1678, 1613, 1584, 1559, 1480, 1421, 1399, 1343, 1320, 1275, 1249, 1196, 1167, 1129, 1075, 1045, 1015, 983, 942, 915; ^1^H NMR (400 MHz, CD_3_CN) δ 7.89 (d, *J* = 7.8 Hz, 1H, 3′-H), 7.56 (dd, *J* = 8.8 Hz, *J* = 2.3 Hz, 1H, 8-H), 7.61–7.49 (m, 2H, 5′-H and 6′-H), 7.38 (d, *J* = 8.8 Hz, 1H, 9-H), 7.23 (ddd, *J* = 9.1 Hz, *J* = 7.8 Hz, *J* = 2.1 Hz, 1H, 4′-H), 7.08 (d, *J* = 2.3 Hz, 1H, 6-H), 4.65 (d, *J* = 10.9 Hz, 1H, 3a-H), 3.76 (d, *J* = 10.9 Hz, 1H, 3b-H), 3.40 (s, 3H, 1-CH_3_); ^13^C NMR (100 MHz, CD_3_CN) δ 172.1 (C = N), 169.7 (C = O), 144.8 (C-1′), 140.4 (C-3′), 135.2 (C-8), 131.9 (C-4′), 131.8 (C-5′ and C-6′), 129.4 (C-6), 124.8 (C-9), 118.3 (C-9a), 117.1 (C-7), 96.7 (C-2′), 57.7 (C-3), 35.0 (1-CH_3_) ppm; HR-MS (ESI+) m/z Calcd for C_16_H_13_BrIN_2_O [M+H^+^] 454.9256, Found 454.9265.

### 7-chloro-2′-iodo-6′-chloro-1,3-dihydro-1-methyl-5-phenyl-2H-1,4-benzodiazepin-2-one (3-I)

To a solution of 7-chloro-6′-chloro-1,3-dihydro-1-methyl-5-phenyl-2H-1,4-benzodiazepin-2-one **3** (10 mg, 0.031 mmol) in CD_3_CN (300 μL), were added Pd(OAc)_2_ (0.7 mg, 0.003 mmol) and N-iodosuccinimide (14 mg, 0.062 mmol). The mixture was stirred at 100 °C under microwave irradiation for 15 minutes. The crude mixture was evaporated, diluted in ethyl acetate (10 mL), and washed with a 2 M aqueous solution of NaOH (5 mL). NMR and LC-MS analysis indicates a 50% conversion of the starting material to the desired compound **3-I**.

### 7-bromo-2′-iodo-4′-methyl-1,3-dihydro-1-methyl-5-phenyl-2H-1,4-benzodiazepin-2-one (4-I)

To a solution of 7-bromo-4′-methyl-1,3-dihydro-1-methyl-5-phenyl-2H-1,4-benzodiazepin-2-one **4** (38 mg, 0.11 mmol) in CD_3_CN (1.l mL), were added Pd(OAc)_2_ (2.5 mg, 0.011 mmol) and N-iodosuccinimide (50 mg, 0.22 mmol). The mixture was stirred at 100 °C under microwave irradiation for 15 minutes. The crude mixture was evaporated, diluted in ethyl acetate (10 mL), and washed with a 2 M aqueous solution of NaOH (5 mL). The residue was purified by flash chromatography (cyclohexane/ethylacetate 1:1) affording 28 mg (54%) of **4-I** and 4 mg (6%) of **4-II**. IR (NaCl, cm^−1^) 3058, 2983, 2919, 2853, 1677, 1612, 1598, 1560, 1478, 1447, 1418, 1398, 1342, 1318, 1275, 1253, 1197, 1168, 1129, 1089, 1075, 1039, 1014, 983, 942, 916; ^1^H NMR (400 MHz, CD_3_CN) δ 7.73 (s, 1H, 3′-H), 7.70 (dd, *J* = 8.8Hz, *J* = 2.4 Hz, 1H, 8-H), 7.43-7.32 (m, 3H, 9-H, 5′-H and 6′-H), 7.06 (d, *J* = 2.3 Hz, 1H, 6-H), 4.62 (d, *J* = 10.7Hz, 1H, 3a-H), 3.79 (d, *J* = 10.7 Hz, 1H, 3b-H), 3.39 (s, 3H, 1-CH_3_), 2.36 (s, 3H, 4′-CH_3_); ^13^C NMR (100 MHz, CD_3_CN) δ 172.0 (C = N), 169.8 (C = O), 144.7 (C-1′), 142.6 (C-4′), 140.8 (C-3′), 135.1 (C-8), 132.1 (C-5a), 131.9 (C-5′ or C-6′), 131.6 (C-6), 130.1 (C-5′ or C-6′), 124.7 (C-9), 118.3 (C-9a), 117.0 (C-7), 96.8 (C-2′), 57.7 (C-3), 35.0 (1-CH_3_), 20.7 (4′-CH_3_); HR-MS (ESI+) m/z Calcd for C_17_H_15_BrIN_2_O [M+H^+^] 468.9412, Found 468.9427.

### 7-bromo-2′-iodo-1,3-dihydro-5-phenyl-2H-1,4-benzodiazepin-2-one (5-I)

To a solution of 7-bromo-1,3-dihydro-5-phenyl-2H-1,4-benzodiazepin-2-one **5** (30 mg, 0.095 mmol) in CD_3_CN (950 μL), were added Pd(OAc)_2_ (2.2 mg, 0.0095 mmol) and N-iodosuccinimide (43 mg, 0.19 mmol). The mixture was stirred at 100 °C under microwave irradiation for 15 minutes. The crude mixture was evaporated, diluted in ethyl acetate (10 mL), and washed with a 2 M aqueous solution of NaOH (5 mL). The residue was purified by flash chromatography (cyclohexane/ethylacetate 1:1) affording 17 mg (41%) of **5-I** and 2 mg (4%) of **5-II**. IR (NaCl, cm^−1^) 3207, 3117, 2927, 2852, 1689, 1617, 1479, 1429, 1382, 1322, 1291, 1255, 1230, 1195, 1164, 1134, 1088, 1047, 1011, 945; ^1^H NMR (400 MHz, (CD_3_)_2_SO) δ 10.82 (s, 1H, 1-H), 7.89 (d, *J* = 7.8 Hz, 1H, 3′-H), 7.71 (dd, *J* = 8.7 Hz, J = 2.3 Hz, 1H, 8-H), 7.52 (dt’, *J* = 7.4 Hz, *J* = 0.8 Hz, 1H, 5′-H), 7.45 (dd, *J* = 7.5 Hz, *J* = 1.4 Hz, 1H, 6′-H), 7.22 (d appt, *J* = 7.8 Hz, *J* = 1.4 Hz, 1H, 4′-H), 7.18 (d, *J* = 8.7 Hz, 1H, 9-H), 6.96 (d, *J* = 2.2 Hz, 1H, 6-H), 4.19 (s, 2H, 3H); ^13^C NMR (100 MHz, (CD_3_)_2_SO) δ 170.7 (C = N), 169.1 (C = O), 143.7 (C-1′), 139.2 (C-5a), 139.1 (C-3′), 134.3 (C-8), 131.4 (C-6), 130.8 (C-4′ and C-6′), 128.1 (C-5′), 123.1 (C-9), 114.3 (C-7), 96.9 (C-2′), 56.8 (C-3); HR-MS (ESI+) m/z Calcd for C_15_H_11_BrIN_2_O [M+H^+^] 440.9099, Found 440.9090.

### 7-chloro-2′-iodo-1,3-dihydro-1-methyl-5-phenyl-2H-1,4-benzodiazepin-2-one (6-I)

To a solution of 7-chloro-1,3-dihydro-1-methyl-5-phenyl-2H-1,4-benzodiazepin-2-one **6** (30 mg, 0.105 mmol) in CD_3_CN (1.05 mL), were added Pd(OAc)_2_ (2.4 mg, 0.010 mmol) and N-iodosuccinimide (47 mg, 0.210 mmol). The mixture was stirred at 100 °C under microwave irradiation for 15 minutes. The crude mixture was evaporated, diluted in ethyl acetate (10 mL), and washed with a 2 M aqueous solution of NaOH (5 mL). The residue was purified by flash chromatography (cyclohexane/ethylacetate 1:1) affording 26 mg (60%) of **6-I** and 2 mg (3%) of **6-II**. IR (NaCl, cm^−1^) 3060, 2988, 2915, 2853, 1678, 1615, 1592, 1582, 1562, 1484, 1447, 1425, 1402, 1346, 1321, 1298, 1276, 1248, 1198, 1169, 1130, 1099, 1076, 1044, 1016, 983, 943, 916; ^1^H NMR (400 MHz, CD_3_CN) δ 7.89 (d, *J* = 7.8 Hz, 1H, 3′-H), 7.58 (dd, *J* = 8.8 Hz, *J* = 2.5 Hz, 1H, 8-H), 7.57–7.49 (m, 2H, 5′-H and 6′-H), 7.44 (d, *J* = 8.8 Hz, 1H, 9-H), 7.22 (d appt, *J* = 7.1 Hz, *J* = 2.2Hz, 1H, 4′-H), 6.94 (d, *J* = 2.5 Hz, 1H, 6-H), 4.65 (d, *J* = 10.8 Hz, 1H, 3a-H), 3.85 (d, *J* = 10.8 Hz, 1H, 3b-H), 3.40 (s, 3H, 1-CH_3_); ^13^C NMR (100 MHz, CD_3_CN) δ 172.1 (C = N), 169.7 (C = O), 144.6 (C-1′), 140.4 (C-3′), 132.3 (C-8), 131.9 (C-4′), 131.8 (C-5′ or C-6′), 131.5 (C-5a), 129.5 (C-7), 129.4 (C-6), 128.8 (C-5′ or C-6′), 124.5 (C-9), 118.3 (C-9a), 96.8 (C-2′), 57.6 (C-3), 35.0 (1-CH_3_); HR-MS (ESI+) m/z Calcd for C_16_H_13_ClIN_2_O [M+H^+^] 410.9761, Found 410.9766.

### Synthesis of 7-bromo-2′-iodo-1,3-dihydro-1-benzyl-5-phenyl-2H-1,4-benzodiazepin-2-one (7-I)

To a solution of 7-bromo-1,3-dihydro-1-benzyl-5-phenyl-2H-1,4-benzodiazepin-2-one (30 mg, 0.074 mmol) in CH_3_CN (740 μL), were added Pd(OAc)_2_ (1.6 mg, 0.0074 mmol) and N-iodosuccinimide (33.5 mg, 0.148 mmol). The mixture stirred at 100 °C under microwave irradiation for 15 minutes under microwave irradiation. The crude mixture was evaporated, diluted in ethyl acetate (10 mL), and washed with a 2M aqueous solution of NaOH (5 mL). The residue was purified by flash chromatography (cyclohexane/ethylacetate 1:1) affording 27 mg (69%) of **7-I** and 3 mg (6%) of **7-II**. IR (KBr, cm^−1^) 3061, 3029, 2995, 2852, 1676, 1612, 1494, 1479, 1454, 1403, 1357, 1319, 1265, 1220, 1185, 1090, 1067, 1015, 938, 882; ^1^H NMR (400 MHz, CD_3_CN) δ 7.90 (d, *J* = 7.9 Hz, 1H, 3′-H), 7.62 (dd, *J* = 8.8 Hz, *J* = 2.1 Hz, 1H, 8-H), 7.50 (t, *J* = 7.5 Hz, 1H), 7.40-7.24 (m, 8H, 8-H, 9-H, 6′-H and Ph), 7.06 (t, *J* = 7.8 Hz, 1H, 4′-H), 7.09 (d, *J* = 2.3 Hz, 1H, 4′-H), 5.24 (d, *J* = 15.9 Hz, 1H, CH_2_-Ph), 5.07 (d, *J* = 15.9 Hz, 1H, CH_2_-Ph), 4.77 (d, *J* = 10.5 Hz, 1H, 3a-H), 4.01 (d, *J* = 10.5 Hz, 1H, 3b-H); ^13^C NMR (100 MHz, CD_3_CN) δ 172.0 (C = N), 168.8 (C = O), 144.5 (C-1′), 140.6 (C-3′), 138.6 (C-5a), 135.3 (C-4), 132.3 (C-6), 132.2 (C-Ph), 131.9 (C-5′ and C-6′), 132.0, 131.9, 129.7, 128.4, 128.3, 124.9 (Ph and C-9), 118.3 (C-9a), 117.6 (C-7), 96.8 (C-2′), 57.8 (C-3), 51.8 (CH_2_-Ph); HR-MS (ESI+) *m/z* Calcd for C_22_H_19_BrIN_2_O [M+H^+^] 530.9569, Found 530.9572.

### 2′-iodo-4′-methyl-1,3-dihydro-1-methyl-5-phenyl-2H-1,4-benzodiazepin-2-one (8-I)

To a solution of 4′-methyl-1,3-dihydro-1-methyl-5-phenyl-2H-1,4-benzodiazepin-2-one **8** (30 mg, 0.11 mmol) in CD_3_CN (1.1 mL), were added Pd(OAc)_2_ (2.5 mg, 0.011 mmol) and N-iodosuccinimide (51 mg, 0.22 mmol). The mixture was stirred at 100 °C under microwave irradiation for 15 minutes. The crude mixture was evaporated, diluted in ethyl acetate (10 mL), and washed with a 2 M aqueous solution of NaOH (5 mL). The residue was purified by flash chromatography (cyclohexane/ethylacetate 1:1) affording 27 mg (61%) of **8-I** and 2 mg (3%) of **8-II**. IR (NaCl, cm^−1^) 3057, 2982, 2920, 2852, 1674, 1598, 1572, 1481, 1447, 1360, 1323, 1280, 1203, 1166, 1128, 1076, 1040, 1010, 984, 939, 915; ^1^H NMR (400 MHz, CD_3_CN) δ 7.73 (s, 1H, 3′-H), 7.56 (d appt, *J* = 7.1 Hz, *J* = 1.4Hz, 1H, 8-H), 7.48-7.30 (m, 3H, 9-H, 5′-H and 6′-H), 7.13 (appt, *J* = 7.2Hz, 1H, 7-H), 7.97 (dd, *J* = 7.8Hz, *J* = 1.3 Hz 1H, 6-H), 4.59 (d, *J* = 10.6 Hz, 1H, 3a-H), 3.78 (d, *J* = 10.6 Hz, 1H, 3b-H), 3.42 (s, 3H, 1-CH_3_), 2.35 (s, 3H, 4′-CH_3_); ^13^C NMR (100 MHz, CD_3_CN) δ 173.4 (C = N), 170.1 (C = O), 145.4 (C-1′), 142.2-142.5 (C-5a), 140.6 (C-3′), 132.4 (C-8), 131.3 (C-5′ or C-6′), 130.3 (C-4′), 129.8-129.6 (C-6, C-5′ or C′6), 124.8 (C-7), 122.5 (C-9), 118.2 (C-9a), 96.8 (C-2′), 57.7 (C-3), 34.9 (1-CH_3_), 20.6 (4′-CH_3_); HR-MS (ESI+) m/z Calcd for C_17_H_16_IN_2_O [M+H^+^] 391.0307, Found 391.0320.

### 2′-bromo-1,3-dihydro-1-methyl-5-phenyl-2H-1,4-benzodiazepin-2-one (1-Br)

To a solution of 1,3-dihydro-1-methyl-5-phenyl-2H-1,4-benzodiazepin-2-one **1** (10 mg, 0.04 mmol) in DMF (400 μL), were added Pd(OAc)_2_ (0.9 mg, 0.004 mmol) and N-bromosuccinimide (36 mg, 0.2 mmol). The mixture was stirred at 100 °C under microwave irradiation for 60 minutes. The crude mixture was diluted in ethyl acetate (10 mL), and washed with a 2 M aqueous solution of NaOH (5 mL). The residue was purified by flash chromatography (cyclohexane/ethylacetate 1:1) affording 5 mg (35%) of **1-Br** and 4 mg (29%) of **3**. IR (NaCl, cm^−1^) 3065, 2990, 2853, 1672, 1611, 1600, 1572, 1490, 1468, 1448, 1361, 1324, 1281, 1202, 1166, 1129, 1077, 1047, 1026, 1010, 985, 939, 915; ^1^H NMR (400 MHz, CD_3_CN) δ 7.62-7.55 (m, 3H, 3′-H, 8-H, 6′-H), 7.51 (d appt, *J* = 7.4 Hz, J = 0.8 Hz, 1H, 5′-H), 7.46 (d, 1H, *J* = 8.1 Hz, 9-H), 7.38 (ddd, *J* = 9.4 Hz, *J* = 7.8 Hz, *J* = 1.8 Hz, 1H, 4′-H), 7.14 (d appt, *J* = 7.9 Hz, *J* = 0.8 Hz, 1H, 7-H), 7.02 (dd, *J* = 7.8 Hz, *J* = 1.4 Hz, 1H, 6-H), 4.61 (d, *J* = 10.6 Hz, 1H, 3a-H), 3.81 (d, *J* = 10.6 Hz, 1H, 3b-H), 3.39 (s, 3H, 1-CH_3_); HR-MS (ESI+) m/z Calcd for C_16_H_14_BrN_2_O [M+H^+^] 329.0290, Found: 329.0286.

### 7-bromo-1,3-dihydro-1-methyl-5-phenyl-2H-1,4-benzodiazepin-2-one (2)

To a solution of 1,3-dihydro-1-methyl-5-phenyl-2H-1,4-benzodiazepin-2-one **1** (10 mg, 0.04 mmol) in DMF (400 μL), was added N-bromosuccinimide (36 mg, 0.2 mmol). The mixture was stirred at 100 °C under microwave irradiation for 60 minutes. The crude mixture was diluted in ethyl acetate (10 mL), and washed with a 2 M aqueous solution of NaOH (5 mL). The residue was purified by flash chromatography (cyclohexane/ethylacetate 1:1) affording 3.5 mg (27%) of **2** and 2.5 mg (25%) of recovered starting material **1**. IR (NaCl, cm^−1^) 3061, 2922, 2852, 1679, 1608, 1480, 1446, 1397, 1339, 1321, 1269, 1198, 1179, 1129, 1090, 1072, 1017, 984, 941, 914; ^1^H NMR (400 MHz, CD_3_CN) δ 7.73 (dd, 1 H, *J* = 2.3Hz, *J* = 8.8 Hz, 8-H), 7.61–7.41 (m, 5H, 2′-H, 3′-H, 4′-H, 5′-H and 6′-H), 7.09–7.36 (m, 2H, 6-H and 9-H), 4.60 (d, *J* = 10.9 Hz, 1 H, 3a-H), 3.78 (d, *J* = 10.7–Hz, 1H, 3b-H), 3.32 (s, 3H, 1-CH_3_); HR-MS (ESI+) m/z Calcd for C_16_H_14_BrN_2_O [M+H^+^] 329.0290, Found 329.0276.

### Crystallographic data collection and structure determination

The data for compound **1-I** were collected at 150(2) K on a Nonius Kappa-CCD area detector diffractometer (Hooft R.W.W: COLLECT: Data Collection Software. Edited by Nonius BV. Delft, The Netherlands; 1998.) using graphite-monochromated Mo-Kα radiation (λ = 0.71073 Å). The unit cell parameters were determined from ten frames, then refined on all data. The data (combination of φ- and ω-scans giving a complete data set up to θ = 30.5° and a minimum redundancy of 4 for 90% of the reflections) were processed with HKL2000^29^. Absorption effects were corrected empirically with the program SCALEPACK.2 The structure was solved by direct methods with SHELXS-97 and refined by full-matrix least-squares on F2 with SHELXL-97^30^. All non-hydrogen atoms were refined with anisotropic displacement parameters. The hydrogen atoms were introduced at calculated positions and were treated as riding atoms with an isotropic displacement parameter equal to 1.2 times that of the parent atom (1.5 for CH_3_).

### Crystal data for compound **1-I**

C_16_H_13_IN_2_O, M = 376.18, monoclinic, space group P21/c, a = 8.1056(3), b = 12.9888(7), c = 13.9601(7) Å, β = 98.725(3)°, V = 1452.74(12) Å^3^, Z = 4, Dc = 1.720 g cm^−3^, μ = 2.201 mm^−1^, F(000) = 736. Refinement of 182 parameters on 4430 independent reflections out of 53320 measured reflections (R_int_ = 0.041) led to R_1_ = 0.032, wR_2_ = 0.085, S = 1.019, Δρmax = 0.82, Δρmin = −0.97 e Å–3.

## Note

^†^Electronic supplementary information (ESI) available: Experimental procedures and characterisation data for all products, including copies of ^1^H and ^13^C NMR spectra and X-ray structural information of **1-I** (CIF). CCDC 1014605.

## Additional Information

**How to cite this article**: Abdelkafi, H. and Cintrat, J.-C. Regioselective Halogenation of 1,4-Benzodiazepinones via CH Activation. *Sci. Rep.* **5**, 12131; doi: 10.1038/srep12131 (2015).

## Supplementary Material

Supplementary Information

## Figures and Tables

**Figure 1:**
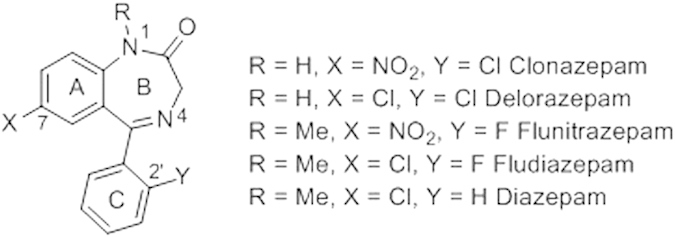
Examples of halogenated 1,4-benzodiazepinones and marketed drugs.

**Table 1:**
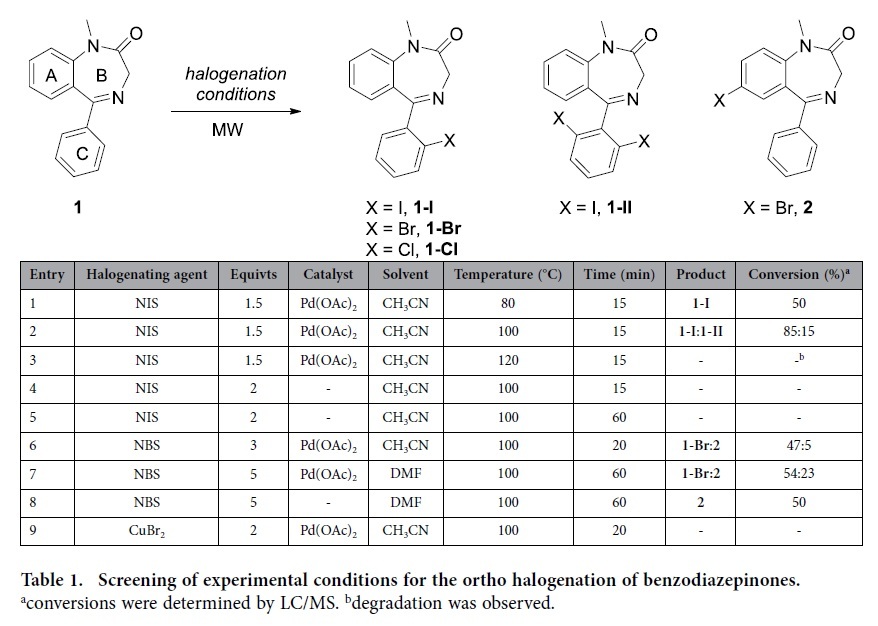
Screening of experimental conditions for the ortho halogenation of benzodiazepinones.

**Table 2:**
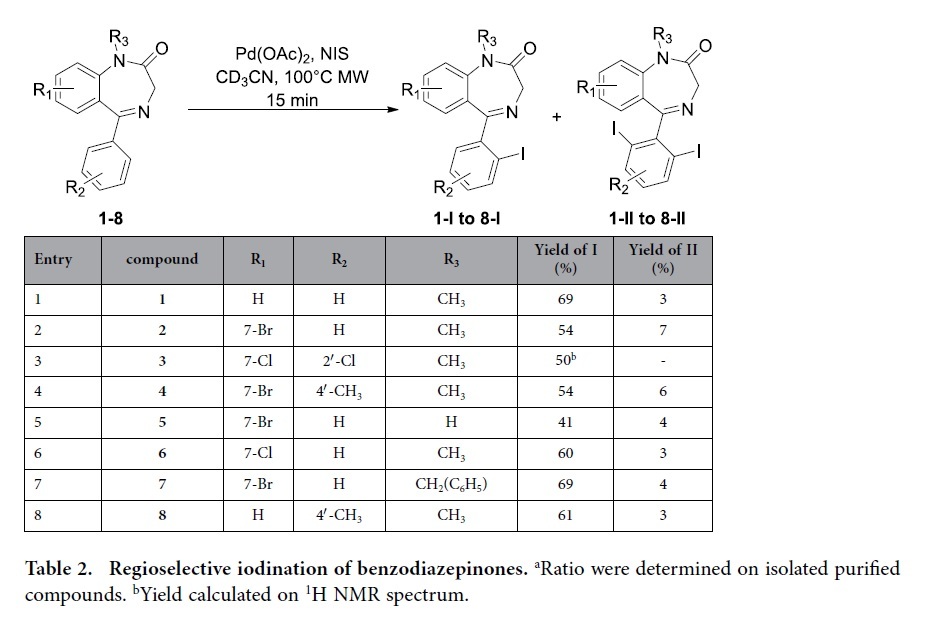
Regioselective iodination of benzodiazepinones.
