# Supplementary material for: Regioselective Halogenation of 1,4-Benzodiazepinones via CH Activation
Source: Sci Rep. 2015 Jul 16;5:12131. doi: 10.1038/srep12131 (PMC4503988; doi:10.1038/srep12131)
Supplement: Supplementary Information [file srep12131-s1.pdf]

Regioselective Halogenation of 1,4-Benzodiazepinones via CH Activation

Hajer Abdelkafi, Jean-Christophe Cintrat*

CEA, iBiTecS, Service de ChimieBioorganique et de Marquage, LabEx LERMIT. 91191 Gif-sur-Yvette (France).

Supplementary Figures Legends

^{1}H and ^{13}C NMR spectra of all new compounds

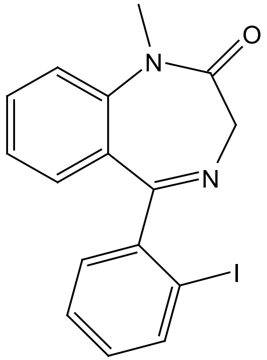

1-I

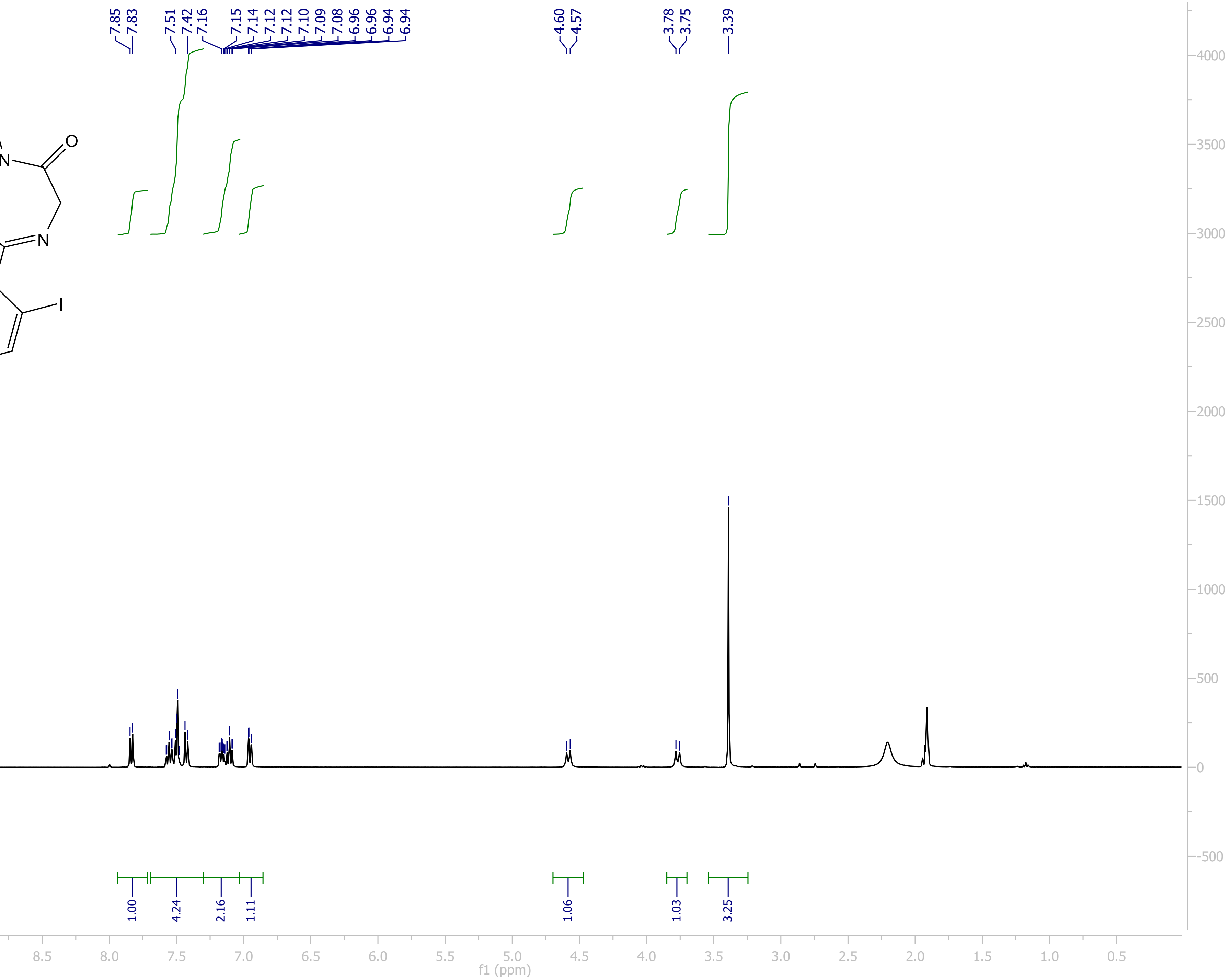
7.85
7.83
7.51
7.42
7.16
7.15
7.14
7.12
7.12
7.10
7.09
7.08
6.96
6.96
6.94
6.94
4.60
4.57
3.78
3.75
3.39
1.00
4.24
2.16
1.11
1.06
1.03
3.25
4000
3500
3000
2500
2000
1500
1000
500
0
-500
10.0
9.5
9.0
8.5
8.0
7.5
7.0
6.5
6.0
5.5
5.0
4.5
4.0
3.5
3.0
2.5
2.0
1.5
1.0
0.5
f1 (ppm)

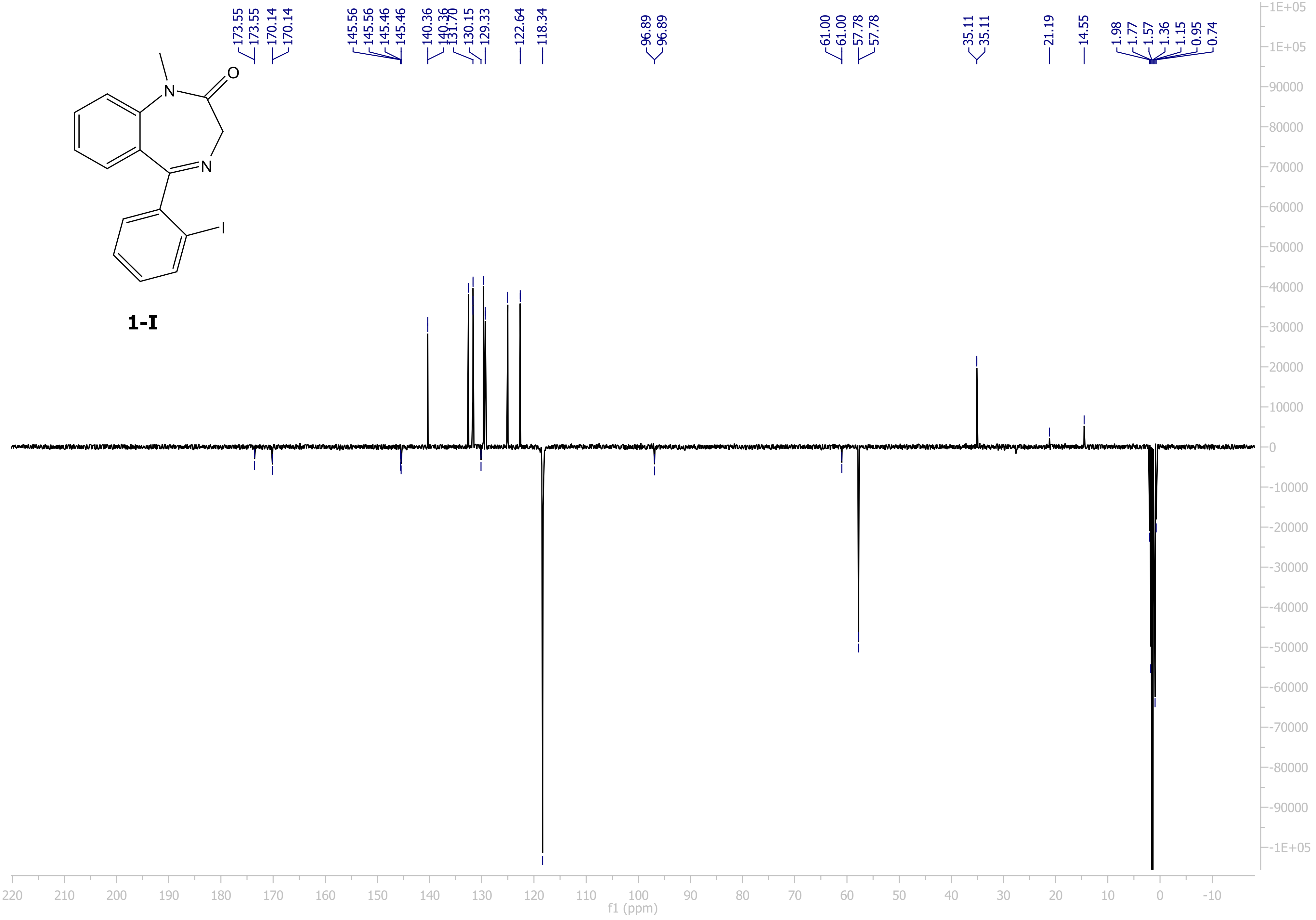
173.55
173.55
170.14
170.14
145.56
145.56
145.46
145.46
140.36
140.36
131.70
130.15
129.33
122.64
118.34
96.89
96.89
61.00
61.00
57.78
57.78
35.11
35.11
21.19
14.55
1.98
1.77
1.57
1.36
1.15
0.95
0.74
O
N
N
I
1-I
f1 (ppm)

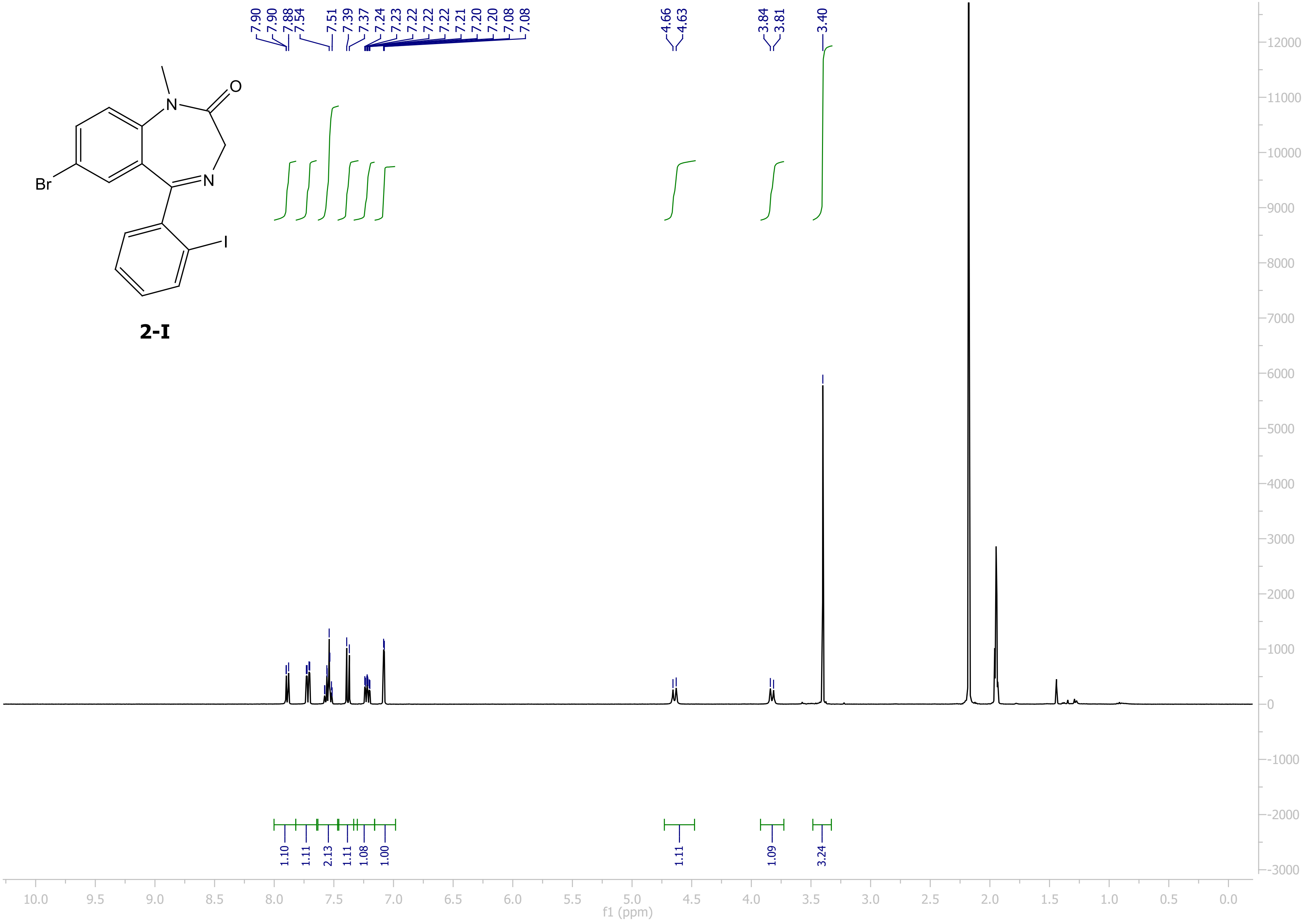
7.90
7.90
7.88
7.54
7.51
7.39
7.37
7.24
7.23
7.22
7.22
7.22
7.21
7.20
7.20
7.08
7.08
4.66
4.63
3.84
3.81
3.40
Br
N
O
N
I
2-I
1.10
1.11
2.13
1.11
1.08
1.00
1.11
1.09
3.24
f1 (ppm)

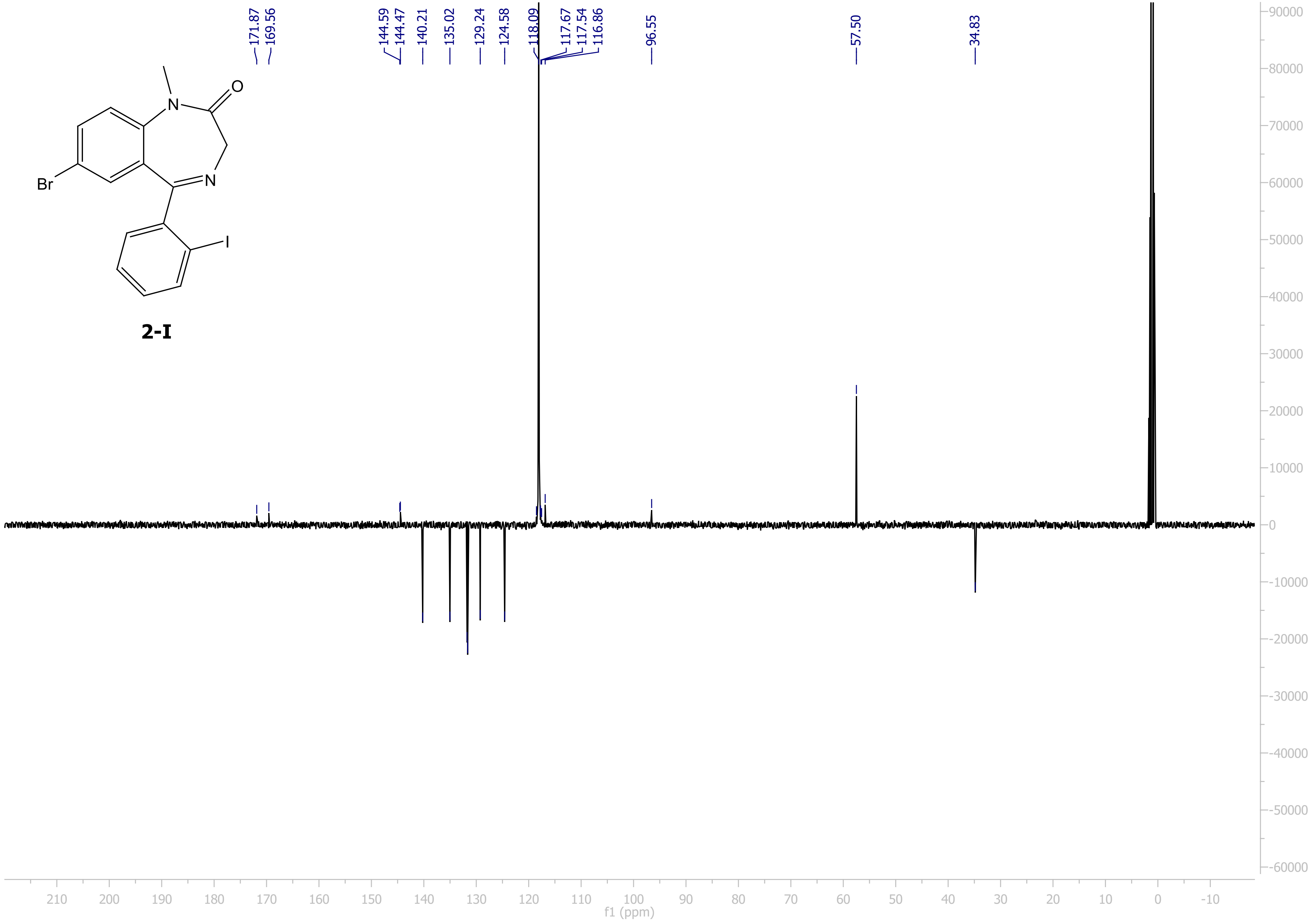
171.87
169.56
144.59
144.47
140.21
135.02
129.24
124.58
118.09
117.67
117.54
116.86
96.55
57.50
34.83
f1 (ppm)

2-I

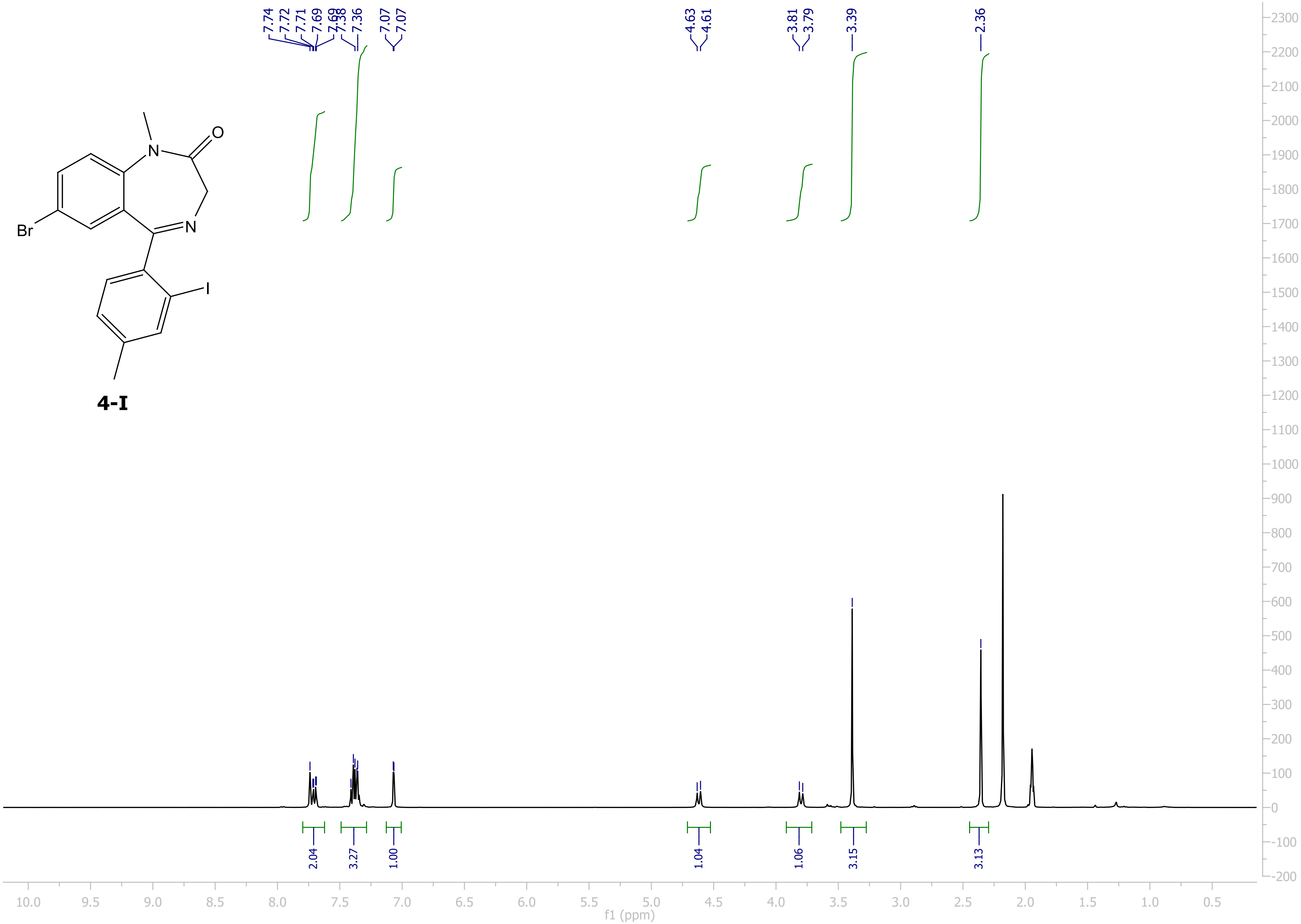

7.74
7.72
7.71
7.69
7.69
7.38
7.36
7.07
7.07
4.63
4.61
3.81
3.79
3.39
2.36
Br
N
O
N
I
4-I
2.04
3.27
1.00
1.04
1.06
3.15
3.13
f1 (ppm)

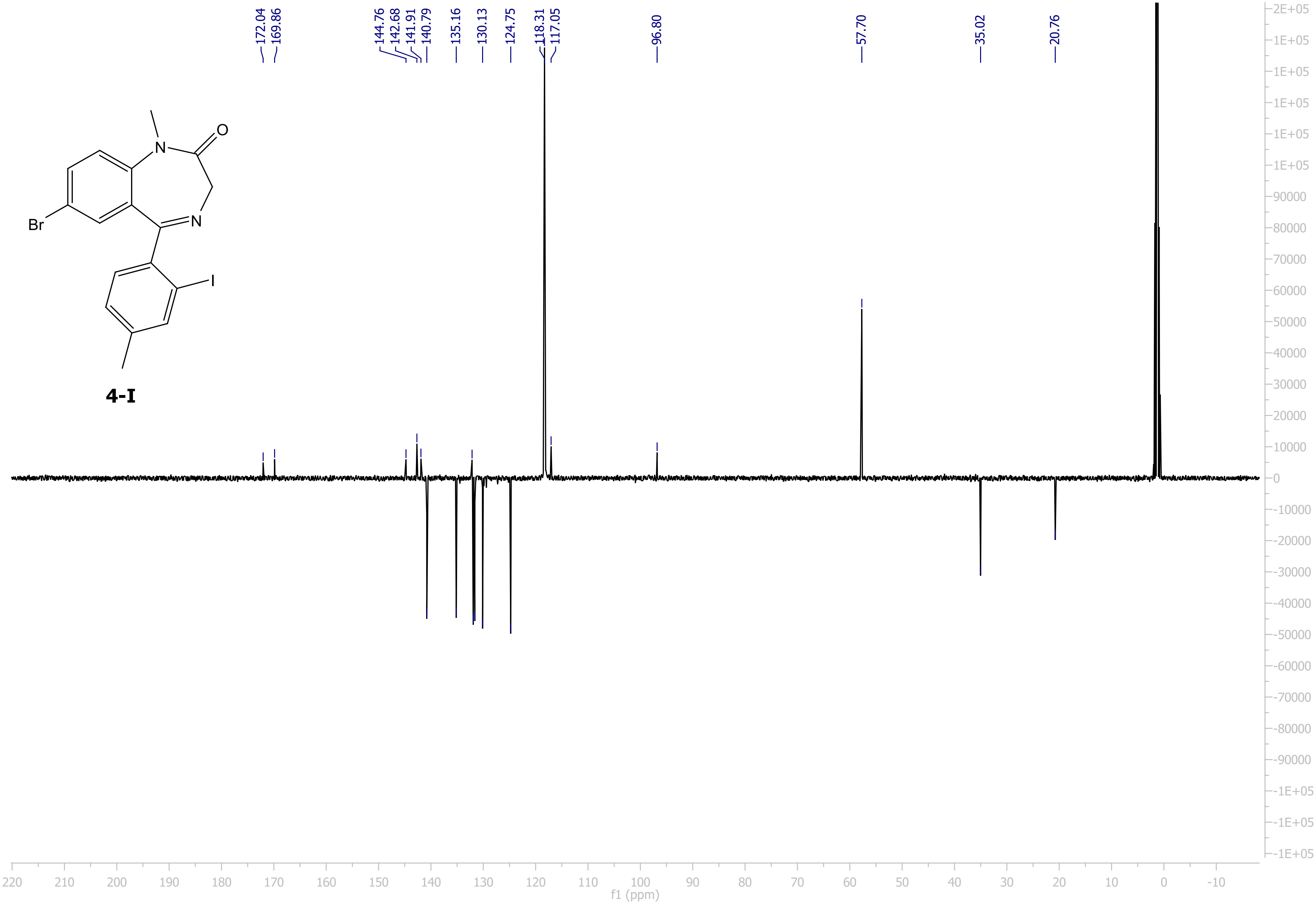

172.04
169.86
144.76
142.68
141.91
140.79
135.16
130.13
124.75
118.31
117.05
96.80
57.70
35.02
20.76
Br
N
O
N
I
4-I
f1 (ppm)
220 210 200 190 180 170 160 150 140 130 120 110 100 90 80 70 60 50 40 30 20 10 0 -10
2E+05
1E+05
1E+05
1E+05
1E+05
1E+05
90000
80000
70000
60000
50000
40000
30000
20000
10000
0
-10000
-20000
-30000
-40000
-50000
-60000
-70000
-80000
-90000
-1E+05
-1E+05
-1E+05

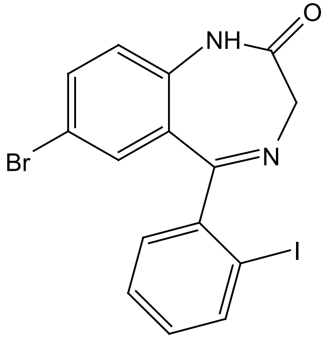
NH
O
Br
N
I

5-I

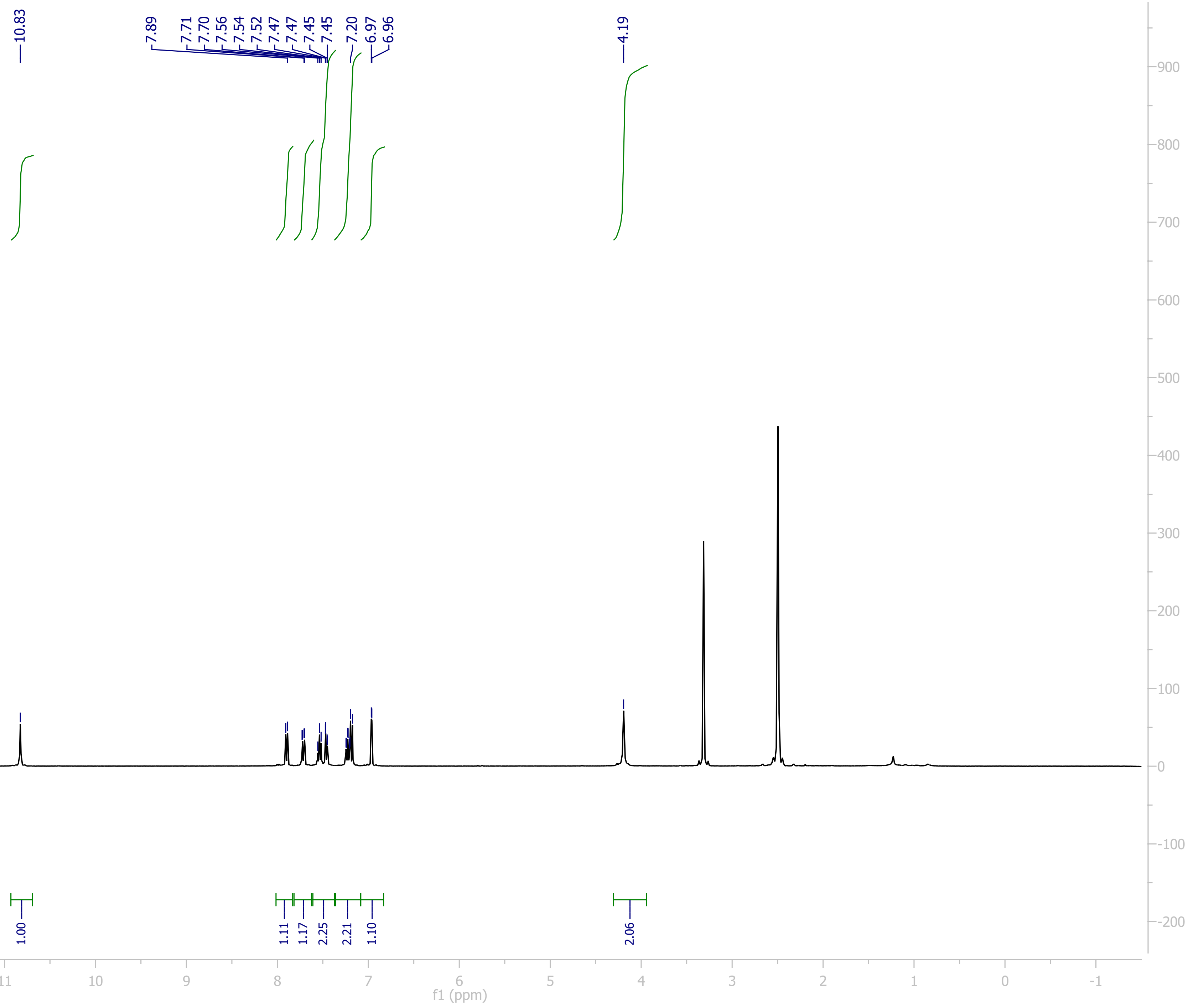
10.83
7.89
7.71
7.70
7.56
7.54
7.52
7.47
7.47
7.45
7.45
7.20
6.97
6.96
4.19
1.00
1.11
1.17
2.25
2.21
1.10
2.06
f1 (ppm)

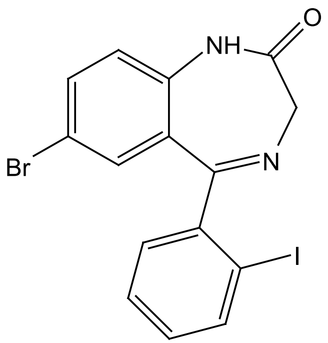

NH
O
Br
N
I

5-I

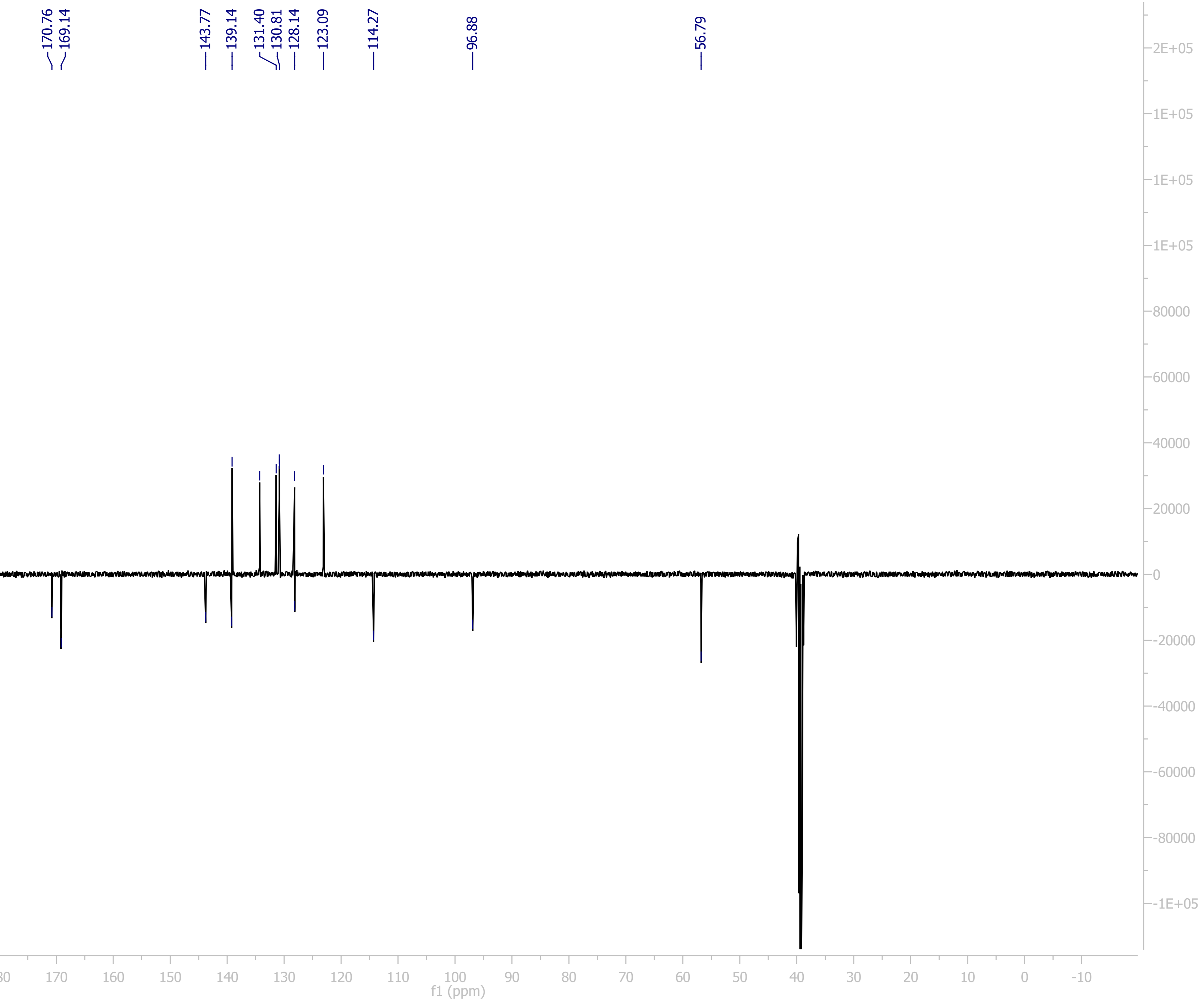

170.76
169.14
143.77
139.14
131.40
130.81
128.14
123.09
114.27
96.88
56.79
2E+05
1E+05
1E+05
1E+05
80000
60000
40000
20000
0
-20000
-40000
-60000
-80000
-1E+05
210
200
190
180
170
160
150
140
130
120
110
100
90
80
70
60
50
40
30
20
10
0
-10
f1 (ppm)

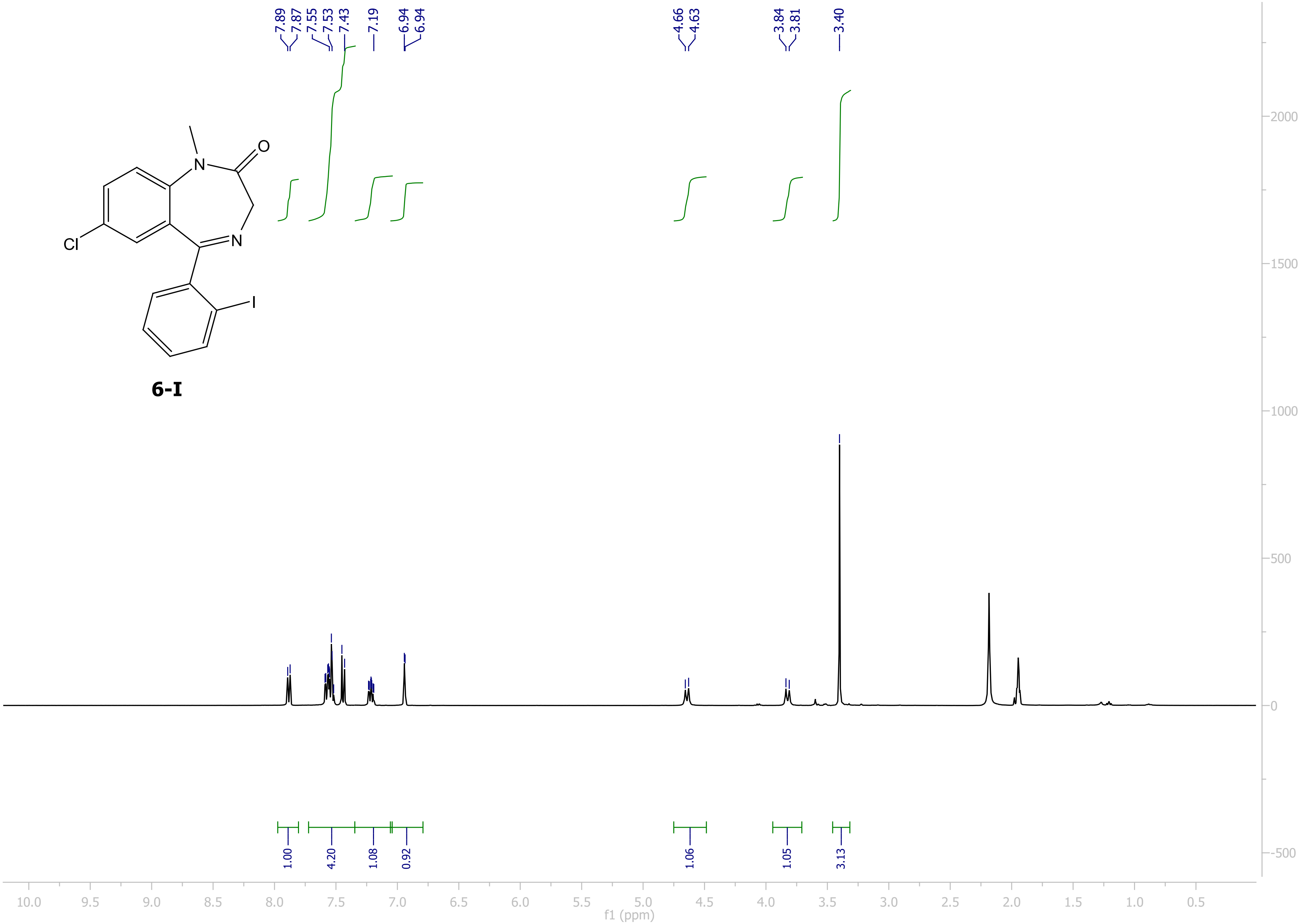

7.89
7.87
7.55
7.53
7.43
7.19
6.94
6.94
4.66
4.63
3.84
3.81
3.40
O
N
N
Cl
I
6-I
1.00
4.20
1.08
0.92
1.06
1.05
3.13
10.0
9.5
9.0
8.5
8.0
7.5
7.0
6.5
6.0
5.5
5.0
4.5
4.0
3.5
3.0
2.5
2.0
1.5
1.0
0.5
f1 (ppm)
2000
1500
1000
500
0
-500

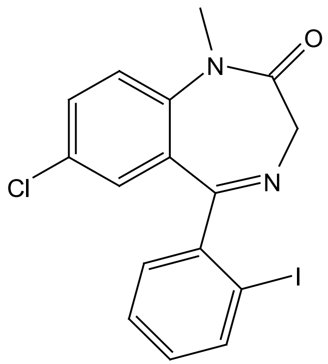

6-I

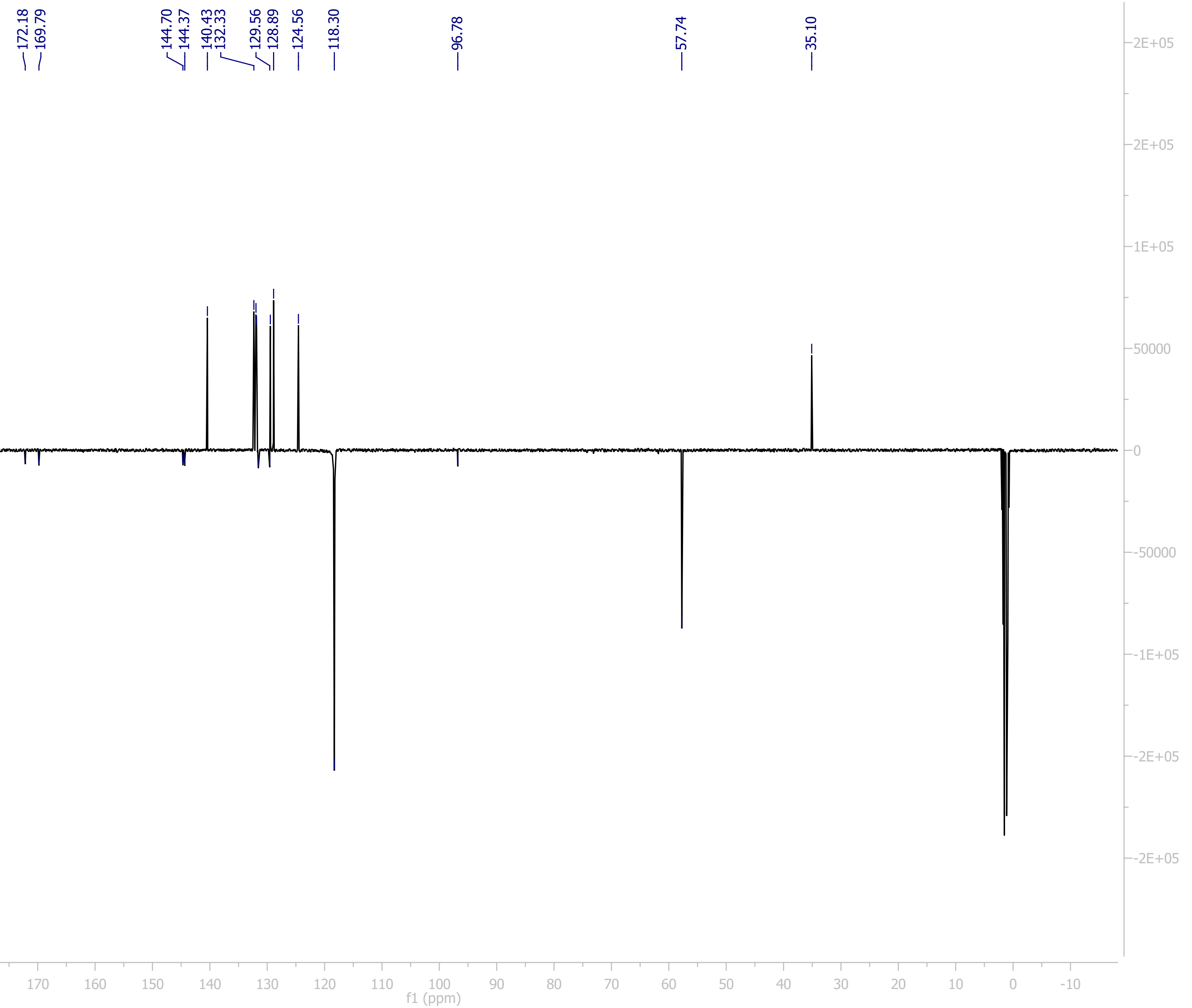

172.18
169.79
144.70
144.37
140.43
132.33
129.56
128.89
124.56
118.30
96.78
57.74
35.10
2E+05
2E+05
1E+05
50000
0
-50000
-1E+05
-2E+05
-2E+05
220
210
200
190
180
170
160
150
140
130
120
110
100
90
80
70
60
50
40
30
20
10
0
-10
f1 (ppm)

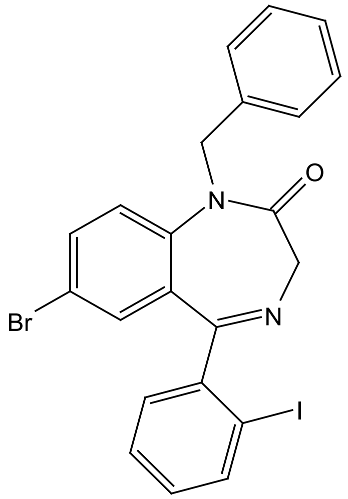

7-I

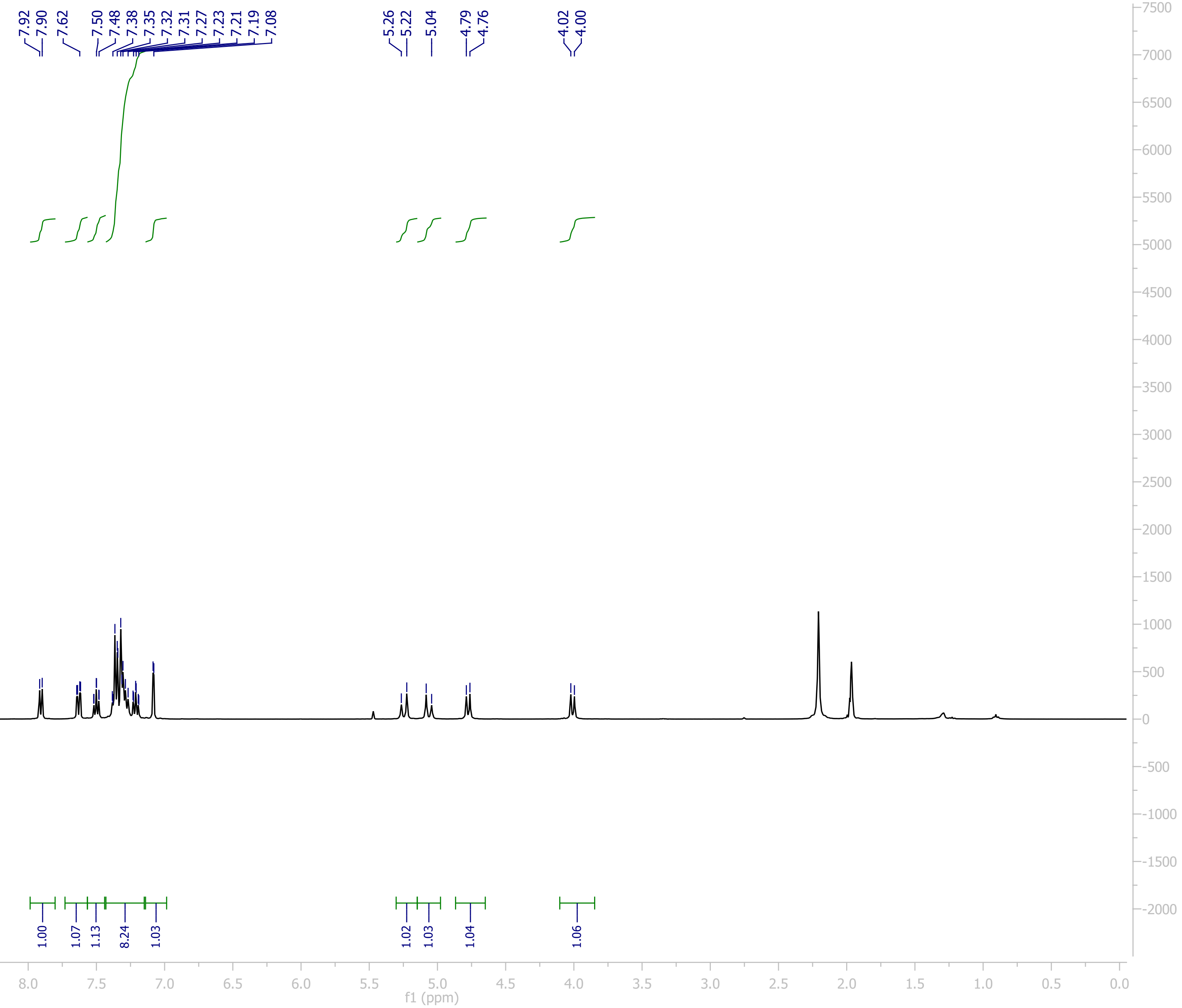
7.92
7.90
7.62
7.50
7.48
7.38
7.35
7.32
7.31
7.27
7.23
7.21
7.19
7.08
5.26
5.22
5.04
4.79
4.76
4.02
4.00
1.00
1.07
1.13
8.24
1.03
1.02
1.03
1.04
1.06
f1 (ppm)

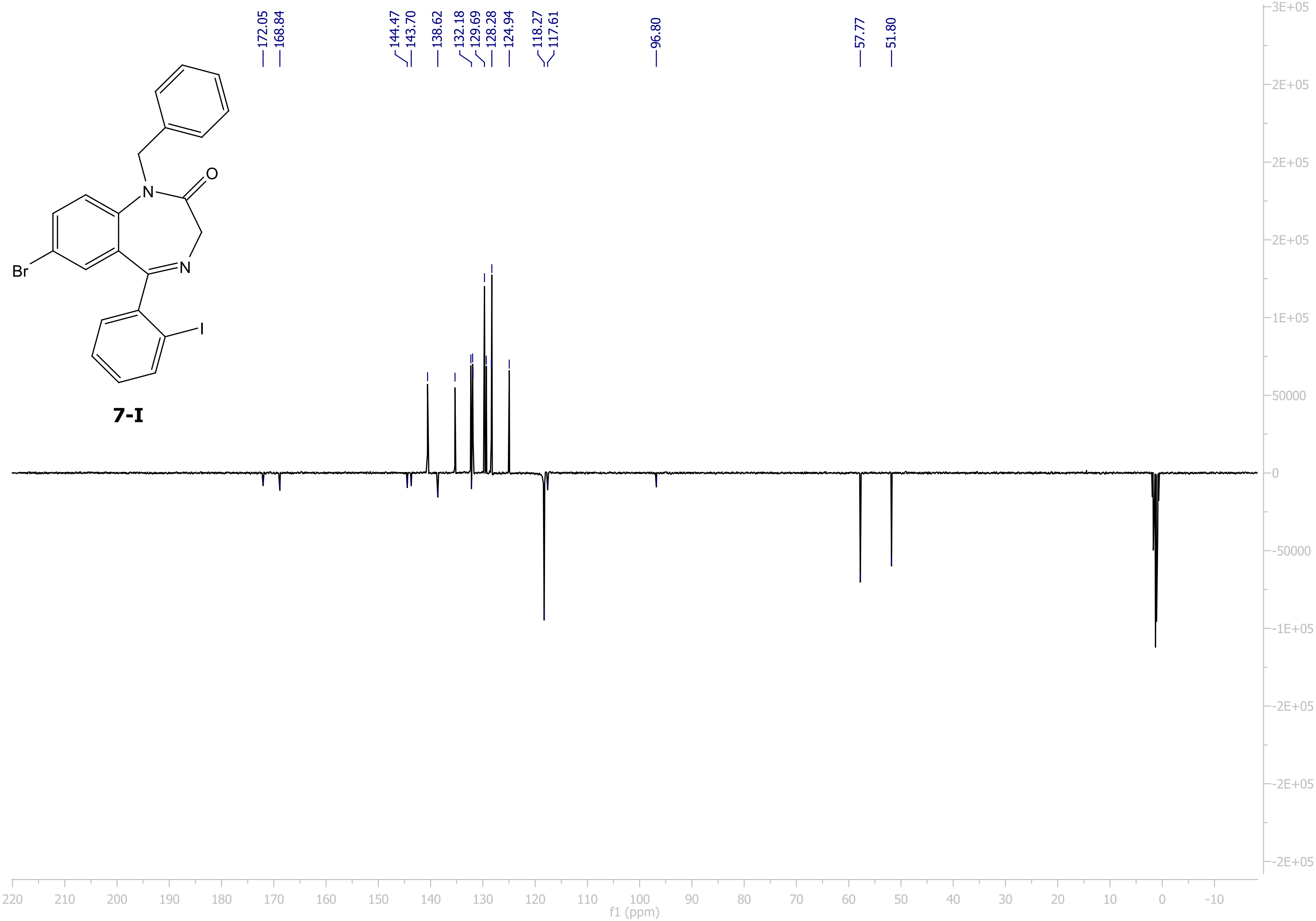
172.05
168.84
144.47
143.70
138.62
132.18
129.69
128.28
124.94
118.27
117.61
96.80
57.77
51.80
Br
N
O
N
I
7-I
3E+05
2E+05
2E+05
2E+05
1E+05
50000
0
-50000
-1E+05
-2E+05
-2E+05
-2E+05
220 210 200 190 180 170 160 150 140 130 120 110 100 90 80 70 60 50 40 30 20 10 0 -10
f1 (ppm)

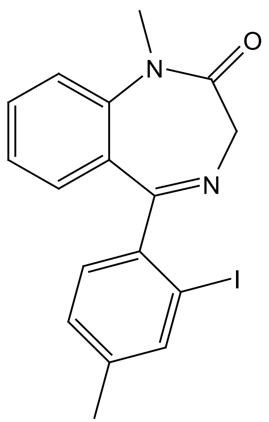

8-I

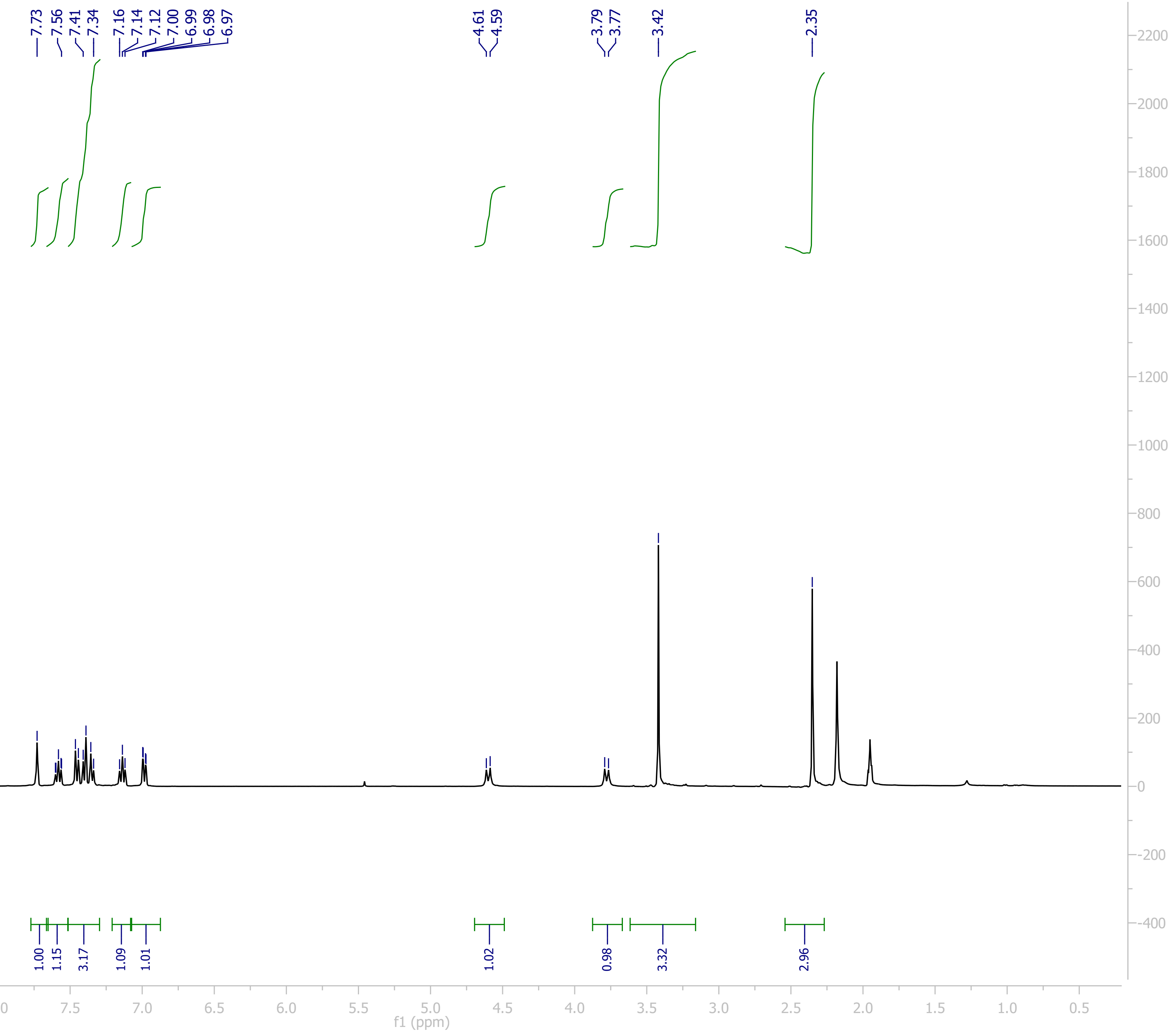
7.73
7.56
7.41
7.34
7.16
7.14
7.12
7.00
6.99
6.98
6.97
4.61
4.59
3.79
3.77
3.42
2.35
1.00
1.15
3.17
1.09
1.01
1.02
0.98
3.32
2.96
9.5
9.0
8.5
8.0
7.5
7.0
6.5
6.0
5.5
5.0
4.5
4.0
3.5
3.0
2.5
2.0
1.5
1.0
0.5
f1 (ppm)
2200
2000
1800
1600
1400
1200
1000
800
600
400
200
0
-200
-400

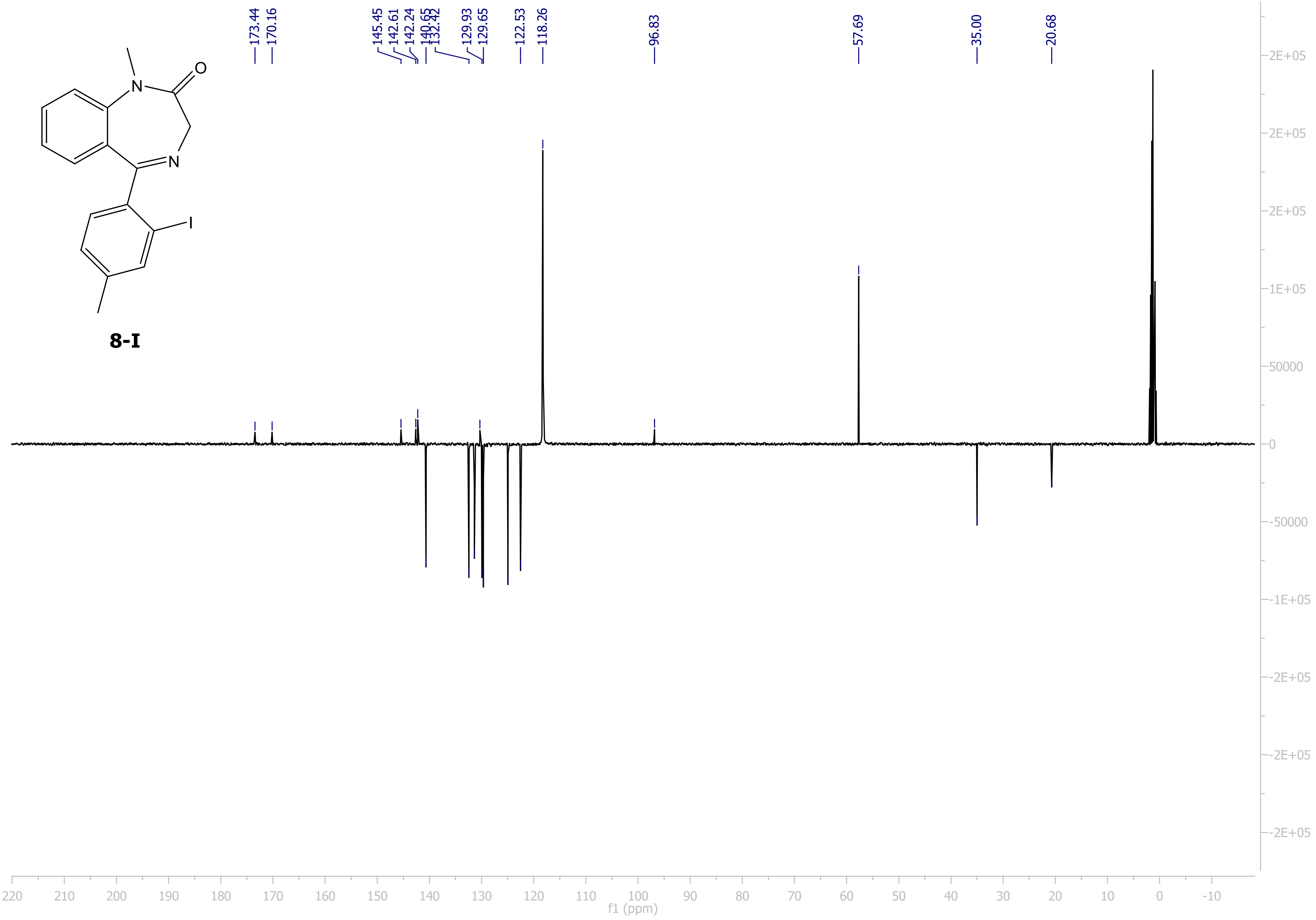

173.44
170.16
145.45
142.61
142.24
140.65
132.42
129.93
129.65
122.53
118.26
96.83
57.69
35.00
20.68
8-I
O
N
N
I
2E+05
2E+05
2E+05
1E+05
50000
0
-50000
-1E+05
-2E+05
-2E+05
-2E+05
220 210 200 190 180 170 160 150 140 130 120 110 100 90 80 70 60 50 40 30 20 10 0 -10
f1 (ppm)

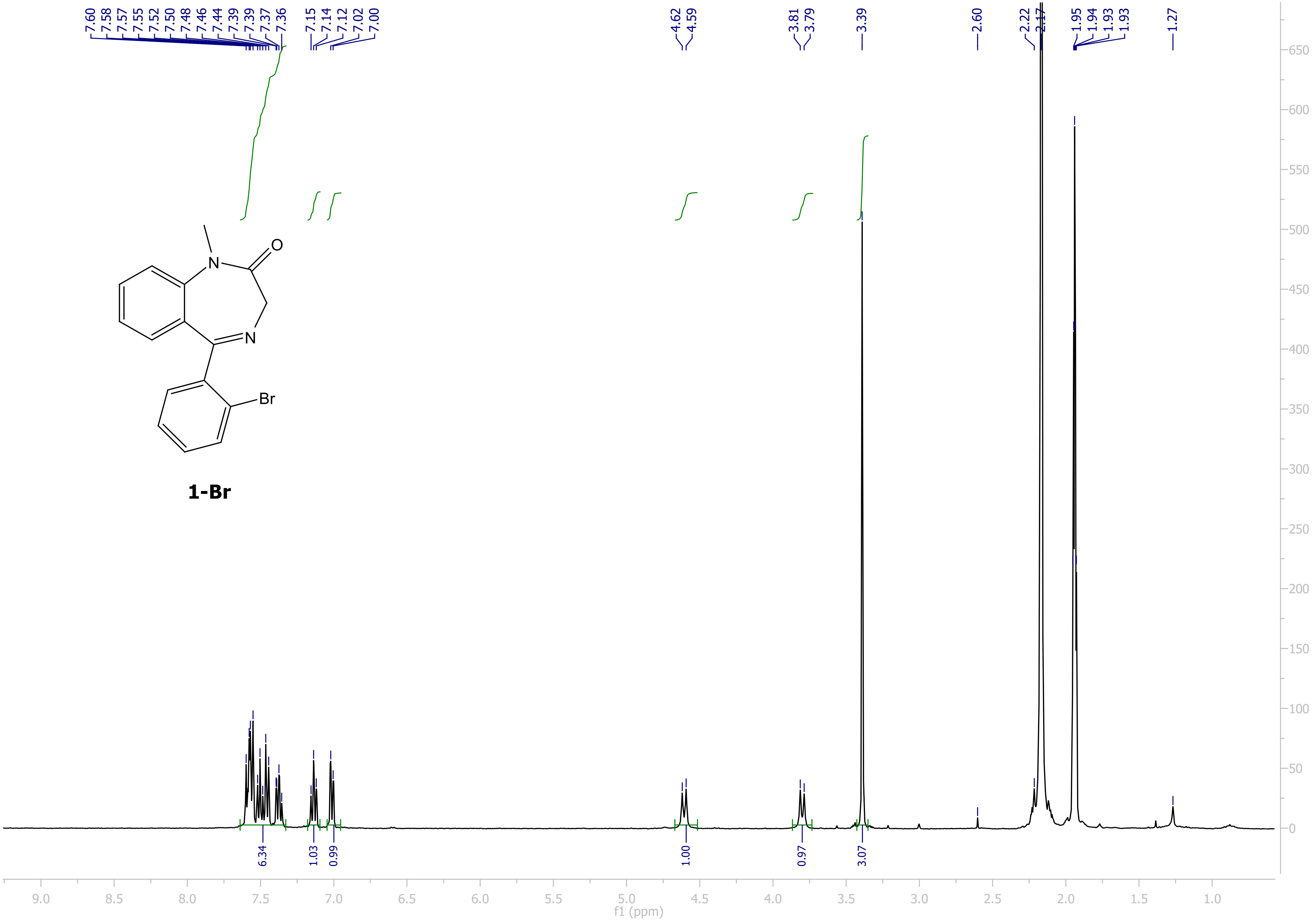
7.60
7.58
7.57
7.55
7.52
7.50
7.48
7.46
7.44
7.39
7.39
7.37
7.36
7.15
7.14
7.12
7.02
7.00
4.62
4.59
3.81
3.79
3.39
2.60
2.22
2.17
1.95
1.94
1.93
1.93
1.27
O
N
N
Br
1-Br
6.34
1.03
0.99
1.00
0.97
3.07
f1 (ppm)

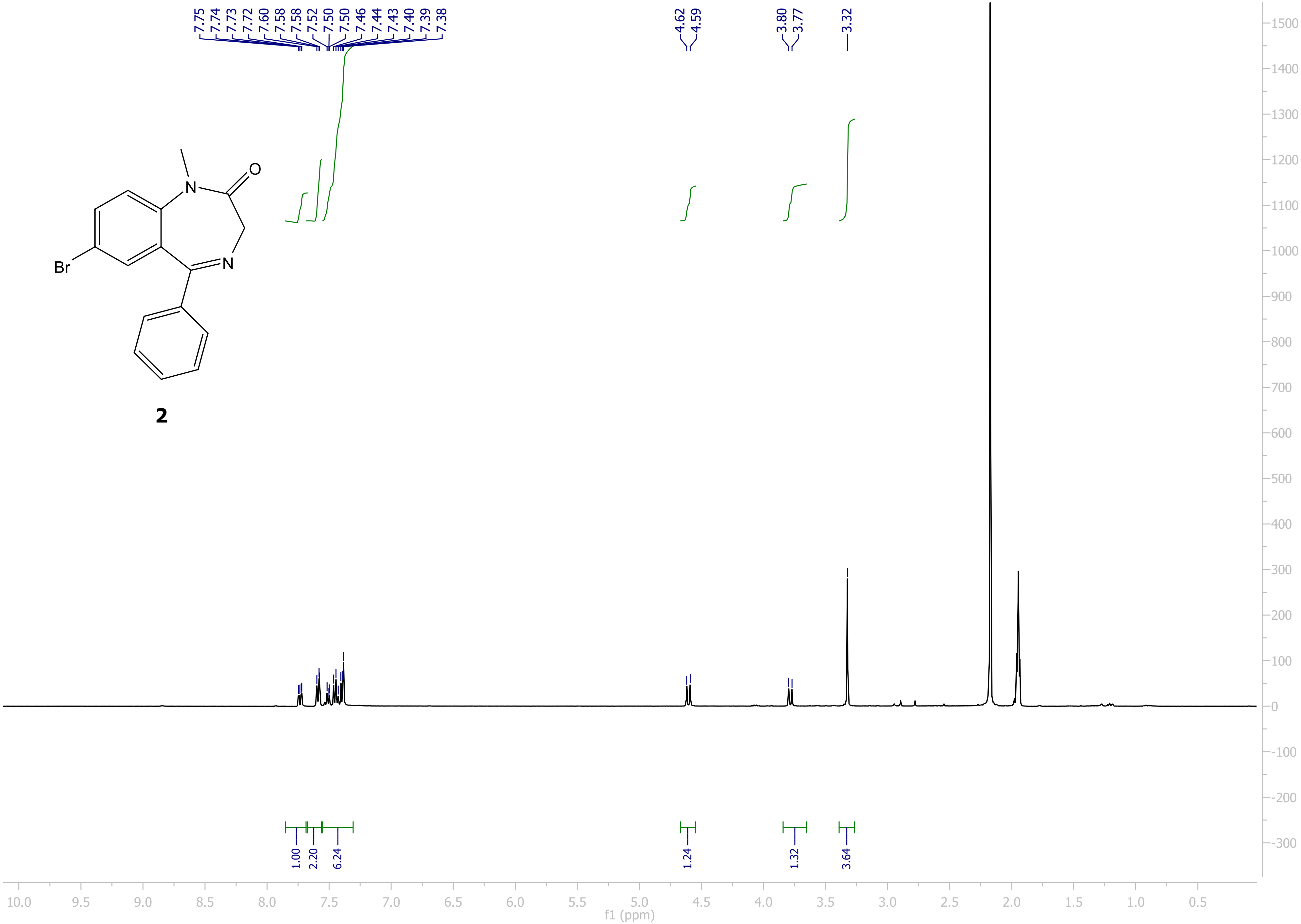

Br
N
O
N
7.75
7.74
7.73
7.72
7.60
7.58
7.52
7.50
7.50
7.46
7.44
7.43
7.40
7.39
7.38
4.62
4.59
3.80
3.77
3.32
1.00
2.20
6.24
1.24
1.32
3.64
f1 (ppm)

2
